# Supplementary material for: The Safety INdEx of Prehospital On Scene Triage (SINEPOST) study: The development and validation of a risk prediction model to support ambulance clinical transport decisions on-scene
Source: PLoS One. 2022 Nov 16;17(11):e0276515. doi: 10.1371/journal.pone.0276515 (PMC9668173; doi:10.1371/journal.pone.0276515)
Supplement: S2 Appendix — (PDF) [file pone.0276515.s002.pdf]

## Appendix S2: List of Emergency Departments included in this study

Barnsley Hospital NHS Foundation Trust

Pinderfields Hospital

St. James's University Hospital

Leeds General Infirmary

Harrogate and District NHS Foundation Trust

Huddersfield Royal Infirmary

Calderdale Royal Hospital

Hull Royal Infirmary

Rotherham NHS Foundation Trust

York Teaching Hospital NHS Foundation Trust

Airedale General Hospital

Doncaster Royal Infirmary

Northern General Hospital

Bradford Royal Infirmary

Dewsbury and District Hospital

Scarborough General Hospital

The James Cook University Hospital
