# Supplementary material for: The Safety INdEx of Prehospital On Scene Triage (SINEPOST) study: The development and validation of a risk prediction model to support ambulance clinical transport decisions on-scene
Source: PLoS One. 2022 Nov 16;17(11):e0276515. doi: 10.1371/journal.pone.0276515 (PMC9668173; doi:10.1371/journal.pone.0276515)
Supplement: S3 Appendix — (PDF) [file pone.0276515.s003.pdf]

Appendix S3: Included variables in the model

	Unavoidable (N=94294)	Avoidable (N=7228)	Overall (N=101522)
**ED (not used as a candidate variable)**			
AIREDALE GENERAL HOSPITAL	3058 (3.2%)	240 (3.3%)	3298 (3.2%)
BARNSLEY DISTRICT GENERAL	5810 (6.2%)	323 (4.5%)	6133 (6.0%)
BRADFORD ROYAL INFIRMARY	6705 (7.1%)	1004 (13.9%)	7709 (7.6%)
CALDERDALE ROYAL HOSPITAL	3865 (4.1%)	242 (3.3%)	4107 (4.0%)
DEWSBURY DISTRICT HOSPITAL	827 (0.9%)	137 (1.9%)	964 (0.9%)
DONCASTER ROYAL INFIRMARY	6258 (6.6%)	420 (5.8%)	6678 (6.6%)
HARROGATE DISTRICT HOSPITAL	2598 (2.8%)	163 (2.3%)	2761 (2.7%)
HUDDERSFIELD ROYAL INFIRMARY	4392 (4.7%)	283 (3.9%)	4675 (4.6%)
HULL ROYAL INFIRMARY	10099 (10.7%)	612 (8.5%)	10711 (10.6%)
JAMES COOK UNIVERSITY HOSPITAL	749 (0.8%)	55 (0.8%)	804 (0.8%)
LEEDS GENERAL INFIRMARY	4839 (5.1%)	263 (3.6%)	5102 (5.0%)
NORTHERN GENERAL HOSPITAL	9793 (10.4%)	929 (12.9%)	10722 (10.6%)
PINDERFIELDS GENERAL HOSPITAL	9481 (10.1%)	764 (10.6%)	10245 (10.1%)
ROTHERHAM DISTRICT GENERAL HOS	5618 (6.0%)	352 (4.9%)	5970 (5.9%)
SCARBOROUGH DISTRICT GENERAL HOSPITAL	4374 (4.6%)	120 (1.7%)	4494 (4.4%)
ST JAMES UNIVERSITY HOSPITAL	8078 (8.6%)	824 (11.4%)	8902 (8.8%)
YORK DISTRICT HOSPITAL	5719 (6.1%)	382 (5.3%)	6101 (6.0%)
Missing	2031 (2.2%)	115 (6.1%)	2146 (2.1%)
**Impression_Psychiatric problems**			
Did not Occur	93634 (99.3%)	6642 (91.9%)	100276 (98.8%)
Occurred	660 (0.7%)	586 (8.1%)	1246 (1.2%)
**cannulation_IV**			
Did not Occur	79660 (84.5%)	6940 (96.0%)	86600 (85.3%)
Occurred	14634 (15.5%)	288 (4.0%)	14922 (14.7%)
**mobililty_SelfMobile**			
Did not Occur	68215 (72.3%)	3436 (47.5%)	71651 (70.6%)
Occurred	26079 (27.7%)	3792 (52.5%)	29871 (29.4%)
**Impression_Allergic reaction/rash**			
Did not Occur	93881 (99.6%)	6971 (96.4%)	100852 (99.3%)
Occurred	413 (0.4%)	257 (3.6%)	670 (0.7%)
**Impression_Cardiac chest pain (ACS)**			
Did not Occur	88507 (93.9%)	7094 (98.1%)	95601 (94.2%)
Occurred	5787 (6.1%)	134 (1.9%)	5921 (5.8%)
**temperature_primary**			
Mean (SD)	37.0 (0.965)	36.8 (0.735)	37.0 (0.952)
Median [Min, Max]	36.9 [31.7, 42.1]	36.8 [33.0, 40.7]	36.9 [31.7, 42.1]
Missing	5935 (6.3%)	796 (11.0%)	6731 (6.6%)
**ecg_monitored_primary**			

Did not Occur	27983 (29.7%)	2988 (41.3%)	30971 (30.5%)
Occurred	51293 (54.4%)	2946 (40.8%)	54239 (53.4%)
Missing	15018 (15.9%)	1294 (17.9%)	16312 (16.1%)
**oxygen_saturations_primary**			
Mean (SD)	95.3 (5.41)	97.1 (2.84)	95.4 (5.29)
Median [Min, Max]	97.0 [11.0, 100]	98.0 [18.0, 100]	97.0 [11.0, 100]
Missing	2543 (2.7%)	329 (4.6%)	2872 (2.8%)
**respiratory_rate_primary**			
Mean (SD)	20.7 (6.30)	18.7 (4.45)	20.5 (6.21)
Median [Min, Max]	18.0 [0, 99.0]	18.0 [0, 96.0]	18.0 [0, 99.0]
Missing	1820 (1.9%)	188 (2.6%)	2008 (2.0%)
**pain_score_primary**			
Mean (SD)	3.10 (3.58)	2.94 (3.50)	3.09 (3.57)
Median [Min, Max]	1.00 [0, 10.0]	0 [0, 10.0]	1.00 [0, 10.0]
Missing	26475 (28.1%)	2073 (28.7%)	28548 (28.1%)
**blood_sugar_reading_primary**			
Mean (SD)	7.43 (3.43)	6.72 (2.72)	7.38 (3.39)
Median [Min, Max]	6.50 [0.600, 35.0]	6.00 [0.400, 33.0]	6.40 [0.400, 35.0]
Missing	23704 (25.1%)	2593 (35.9%)	26297 (25.9%)
**manual_pulse_rate_primary**			
Mean (SD)	89.2 (22.3)	88.1 (18.2)	89.1 (22.0)
Median [Min, Max]	86.0 [5.00, 220]	87.0 [6.00, 220]	86.0 [5.00, 220]
Missing	2186 (2.3%)	309 (4.3%)	2495 (2.5%)
**obs_supplimental_oxygen_subsequent**			
Did not Occur	56181 (59.6%)	4030 (55.8%)	60211 (59.3%)
Occurred	12364 (13.1%)	149 (2.1%)	12513 (12.3%)
Missing	25749 (27.3%)	3049 (42.2%)	28798 (28.4%)
**Impression_Head injury**			
Did not Occur	92710 (98.3%)	6937 (96.0%)	99647 (98.2%)
Occurred	1584 (1.7%)	291 (4.0%)	1875 (1.8%)
**Impression_Pain - back non-traumatic**			
Did not Occur	92414 (98.0%)	6882 (95.2%)	99296 (97.8%)
Occurred	1880 (2.0%)	346 (4.8%)	2226 (2.2%)
**manual_pulse_rate_subsequent**			
Mean (SD)	87.8 (22.0)	86.5 (17.3)	87.7 (21.8)
Median [Min, Max]	85.0 [9.00, 220]	85.0 [37.0, 188]	85.0 [9.00, 220]
Missing	27444 (29.1%)	3166 (43.8%)	30610 (30.2%)
**bp_diastolic_subsequent**			
Mean (SD)	81.4 (17.3)	85.2 (15.3)	81.6 (17.2)
Median [Min, Max]	81.0 [0, 200]	85.0 [32.0, 168]	81.0 [0, 200]
Missing	29222 (31.0%)	3263 (45.1%)	32485 (32.0%)
**Impression_Minor cuts & bruising**			

Did not Occur	94126 (99.8%)	7142 (98.8%)	101268 (99.7%)
Occurred	168 (0.2%)	86 (1.2%)	254 (0.3%)
**drug_Oxygen**			
Did not Occur	82346 (87.3%)	7077 (97.9%)	89423 (88.1%)
Occurred	11948 (12.7%)	151 (2.1%)	12099 (11.9%)
**respiratory_rate_subsequent**			
Mean (SD)	20.3 (5.94)	18.3 (3.52)	20.2 (5.84)
Median [Min, Max]	18.0 [0, 99.0]	18.0 [1.00, 81.0]	18.0 [0, 99.0]
Missing	26569 (28.2%)	3097 (42.8%)	29666 (29.2%)
**Impression_Unable to cope**			
Did not Occur	93990 (99.7%)	7137 (98.7%)	101127 (99.6%)
Occurred	304 (0.3%)	91 (1.3%)	395 (0.4%)
**drug_Aspirin**			
Did not Occur	90409 (95.9%)	7164 (99.1%)	97573 (96.1%)
Occurred	3885 (4.1%)	64 (0.9%)	3949 (3.9%)
**Impression_Abdominal pain**			
Did not Occur	86900 (92.2%)	6780 (93.8%)	93680 (92.3%)
Occurred	7394 (7.8%)	448 (6.2%)	7842 (7.7%)
**bp_systolic_primary**			
Mean (SD)	143 (28.3)	143 (24.4)	143 (28.1)
Median [Min, Max]	142 [0, 265]	140 [1.00, 288]	142 [0, 288]
Missing	2991 (3.2%)	388 (5.4%)	3379 (3.3%)
**bp_diastolic_primary**			
Mean (SD)	82.9 (17.7)	86.4 (15.6)	83.2 (17.6)
Median [Min, Max]	83.0 [0, 200]	86.0 [4.00, 182]	83.0 [0, 200]
Missing	3114 (3.3%)	397 (5.5%)	3511 (3.5%)
**PulseInt**			
Mean (SD)	-2.14 (11.2)	-2.68 (9.52)	-2.18 (11.1)
Median [Min, Max]	-1.00 [-149, 144]	-2.00 [-127, 94.0]	-1.00 [-149, 144]
Missing	27964 (29.7%)	3192 (44.2%)	31156 (30.7%)
**Impression_Wound Closure**			
Did not Occur	94206 (99.9%)	7193 (99.5%)	101399 (99.9%)
Occurred	88 (0.1%)	35 (0.5%)	123 (0.1%)
**epr_news_score_0**			
Did not Occur	65881 (69.9%)	4103 (56.8%)	69984 (68.9%)
Occurred	20807 (22.1%)	2194 (30.4%)	23001 (22.7%)
Missing	7606 (8.1%)	931 (12.9%)	8537 (8.4%)
**bp_systolic_subsequent**			
Mean (SD)	141 (28.0)	140 (24.2)	141 (27.8)
Median [Min, Max]	139 [0, 282]	138 [59.0, 242]	139 [0, 282]
Missing	29145 (30.9%)	3262 (45.1%)	32407 (31.9%)
**Impression_Drug overdose**			
Did not Occur	92781 (98.4%)	7068 (97.8%)	99849 (98.4%)

Occurred	1513 (1.6%)	160 (2.2%)	1673 (1.6%)
**Impression_Stroke FAST positive**			
Did not Occur	92749 (98.4%)	7204 (99.7%)	99953 (98.5%)
Occurred	1545 (1.6%)	24 (0.3%)	1569 (1.5%)
**Impression_Eye injury/eye problem**			
Did not Occur	94200 (99.9%)	7187 (99.4%)	101387 (99.9%)
Occurred	94 (0.1%)	41 (0.6%)	135 (0.1%)
**Impression_Alcohol related**			
Did not Occur	93893 (99.6%)	7114 (98.4%)	101007 (99.5%)
Occurred	401 (0.4%)	114 (1.6%)	515 (0.5%)
**Impression_Fracture/possible fracture**			
Did not Occur	91981 (97.5%)	7174 (99.3%)	99155 (97.7%)
Occurred	2313 (2.5%)	54 (0.7%)	2367 (2.3%)
**Impression_Haemorrhage/lacerations**			
Did not Occur	93896 (99.6%)	7135 (98.7%)	101031 (99.5%)
Occurred	398 (0.4%)	93 (1.3%)	491 (0.5%)
**SysBPInt**			
Mean (SD)	-2.36 (17.7)	-2.84 (14.6)	-2.39 (17.5)
Median [Min, Max]	-1.00 [-182, 239]	-2.00 [-85.0, 147]	-1.00 [-182, 239]
Missing	29901 (31.7%)	3300 (45.7%)	33201 (32.7%)
**Impression_Collapse-reason unknown**			
Did not Occur	92200 (97.8%)	7168 (99.2%)	99368 (97.9%)
Occurred	2094 (2.2%)	60 (0.8%)	2154 (2.1%)
**O2Int**			
Mean (SD)	1.42 (5.12)	0.146 (3.19)	1.34 (5.04)
Median [Min, Max]	0 [-84.0, 86.0]	0 [-81.0, 80.0]	0 [-84.0, 86.0]
Missing	27728 (29.4%)	3176 (43.9%)	30904 (30.4%)
**Impression_Cardiac Arrhythmia**			
Did not Occur	92568 (98.2%)	7194 (99.5%)	99762 (98.3%)
Occurred	1726 (1.8%)	34 (0.5%)	1760 (1.7%)
**drug_GTN**			
Did not Occur	90832 (96.3%)	7178 (99.3%)	98010 (96.5%)
Occurred	3462 (3.7%)	50 (0.7%)	3512 (3.5%)
**Impression_Minor injuries - other**			
Did not Occur	93776 (99.5%)	7130 (98.6%)	100906 (99.4%)
Occurred	518 (0.5%)	98 (1.4%)	616 (0.6%)
**DiaBPInt**			
Mean (SD)	-1.46 (13.5)	-1.83 (11.4)	-1.48 (13.4)
Median [Min, Max]	-1.00 [-127, 135]	-1.00 [-73.0, 84.0]	-1.00 [-127, 135]
Missing	30059 (31.9%)	3307 (45.8%)	33366 (32.9%)
**psyc_AVPU_Confusion**			
Did not Occur	90490 (96.0%)	7133 (98.7%)	97623 (96.2%)
Occurred	3201 (3.4%)	63 (0.9%)	3264 (3.2%)

Missing	603 (0.6%)	32 (0.4%)	635 (0.6%)
**oxygen_saturations_subsequent**			
Mean (SD)	96.3 (3.30)	97.2 (2.92)	96.4 (3.28)
Median [Min, Max]	97.0 [14.0, 100]	98.0 [17.0, 100]	97.0 [14.0, 100]
Missing	27171 (28.8%)	3146 (43.5%)	30317 (29.9%)
**Location_Domestic Address**			
Did not Occur	15303 (16.2%)	1106 (15.3%)	16409 (16.2%)
Occurred	68004 (72.1%)	5281 (73.1%)	73285 (72.2%)
Missing	10987 (11.7%)	841 (11.6%)	11828 (11.7%)
**drug_Morphine.Sulphate**			
Did not Occur	89586 (95.0%)	7129 (98.6%)	96715 (95.3%)
Occurred	4708 (5.0%)	99 (1.4%)	4807 (4.7%)
**Impression_Burns**			
Did not Occur	94243 (99.9%)	7204 (99.7%)	101447 (99.9%)
Occurred	51 (0.1%)	24 (0.3%)	75 (0.1%)
**Impression_No injury or illness**			
Did not Occur	94126 (99.8%)	7183 (99.4%)	101309 (99.8%)
Occurred	168 (0.2%)	45 (0.6%)	213 (0.2%)
**drug_Entonox**			
Did not Occur	89453 (94.9%)	6890 (95.3%)	96343 (94.9%)
Occurred	4841 (5.1%)	338 (4.7%)	5179 (5.1%)
**Impression_Panic attack**			
Did not Occur	94078 (99.8%)	7151 (98.9%)	101229 (99.7%)
Occurred	216 (0.2%)	77 (1.1%)	293 (0.3%)
**drug_Chlorphenamine**			
Did not Occur	94123 (99.8%)	7159 (99.0%)	101282 (99.8%)
Occurred	171 (0.2%)	69 (1.0%)	240 (0.2%)
**Impression_Headache**			
Did not Occur	93387 (99.0%)	7050 (97.5%)	100437 (98.9%)
Occurred	907 (1.0%)	178 (2.5%)	1085 (1.1%)
**Impression_Seizures (non-EP)**			
Did not Occur	93531 (99.2%)	7198 (99.6%)	100729 (99.2%)
Occurred	763 (0.8%)	30 (0.4%)	793 (0.8%)
**epr_news_score_1**			
Did not Occur	69887 (74.1%)	4518 (62.5%)	74405 (73.3%)
Occurred	16801 (17.8%)	1779 (24.6%)	18580 (18.3%)
Missing	7606 (8.1%)	931 (12.9%)	8537 (8.4%)
**Impression_Vomiting**			
Did not Occur	93084 (98.7%)	7167 (99.2%)	100251 (98.7%)
Occurred	1210 (1.3%)	61 (0.8%)	1271 (1.3%)
**Impression_Catheter problems**			
Did not Occur	93956 (99.6%)	7181 (99.3%)	101137 (99.6%)
Occurred	338 (0.4%)	47 (0.7%)	385 (0.4%)

**Impression_Pain - other**			
Did not Occur	84576 (89.7%)	6293 (87.1%)	90869 (89.5%)
Occurred	9718 (10.3%)	935 (12.9%)	10653 (10.5%)
**avpu_score_subsequent_1**			
Did not Occur	68458 (72.6%)	4199 (58.1%)	72657 (71.6%)
Occurred	450 (0.5%)	2 (0.0%)	452 (0.4%)
Missing	25386 (26.9%)	3027 (41.9%)	28413 (28.0%)
**RRInt**			
Mean (SD)	-0.898 (3.94)	-0.744 (3.50)	-0.889 (3.91)
Median [Min, Max]	0 [-83.0, 84.0]	0 [-76.0, 57.0]	0 [-83.0, 84.0]
Missing	26941 (28.6%)	3114 (43.1%)	30055 (29.6%)
**drug_Adrenaline.1:1000**			
Did not Occur	94158 (99.9%)	7223 (99.9%)	101381 (99.9%)
Occurred	136 (0.1%)	5 (0.1%)	141 (0.1%)
**Impression_Haematemesis**			
Did not Occur	93671 (99.3%)	7204 (99.7%)	100875 (99.4%)
Occurred	623 (0.7%)	24 (0.3%)	647 (0.6%)
**Location_Care Home**			
Did not Occur	75693 (80.3%)	6015 (83.2%)	81708 (80.5%)
Occurred	7614 (8.1%)	372 (5.1%)	7986 (7.9%)
Missing	10987 (11.7%)	841 (11.6%)	11828 (11.7%)
**avpu_score_primary_4**			
Did not Occur	89568 (95.0%)	7033 (97.3%)	96601 (95.2%)
Occurred	3434 (3.6%)	66 (0.9%)	3500 (3.4%)
Missing	1292 (1.4%)	129 (1.8%)	1421 (1.4%)
**obs_supplimental_oxygen_primary**			
Did not Occur	88112 (93.4%)	6998 (96.8%)	95110 (93.7%)
Occurred	4560 (4.8%)	78 (1.1%)	4638 (4.6%)
Missing	1622 (1.7%)	152 (2.1%)	1774 (1.7%)
**Location_Other**			
Did not Occur	78858 (83.6%)	6067 (83.9%)	84925 (83.7%)
Occurred	4449 (4.7%)	320 (4.4%)	4769 (4.7%)
Missing	10987 (11.7%)	841 (11.6%)	11828 (11.7%)
**Impression_Falls**			
Did not Occur	87481 (92.8%)	6876 (95.1%)	94357 (92.9%)
Occurred	6813 (7.2%)	352 (4.9%)	7165 (7.1%)
**Impression_Other medical condition**			
Did not Occur	89214 (94.6%)	6742 (93.3%)	95956 (94.5%)
Occurred	5080 (5.4%)	486 (6.7%)	5566 (5.5%)
**Impression_Choking**			
Did not Occur	94216 (99.9%)	7211 (99.8%)	101427 (99.9%)
Occurred	78 (0.1%)	17 (0.2%)	95 (0.1%)
**epr_nok_named**			

Did not Occur	7556 (8.0%)	706 (9.8%)	8262 (8.1%)
Occurred	86738 (92.0%)	6522 (90.2%)	93260 (91.9%)
**avpu_score_subsequent_3**			
Did not Occur	68517 (72.7%)	4193 (58.0%)	72710 (71.6%)
Occurred	391 (0.4%)	8 (0.1%)	399 (0.4%)
Missing	25386 (26.9%)	3027 (41.9%)	28413 (28.0%)
**Impression_Dental**			
Did not Occur	94261 (100.0%)	7213 (99.8%)	101474 (100.0%)
Occurred	33 (0.0%)	15 (0.2%)	48 (0.0%)
**Impression_Bleeding PR**			
Did not Occur	93551 (99.2%)	7192 (99.5%)	100743 (99.2%)
Occurred	743 (0.8%)	36 (0.5%)	779 (0.8%)
**epr_news_score_2**			
Did not Occur	76161 (80.8%)	5369 (74.3%)	81530 (80.3%)
Occurred	10527 (11.2%)	928 (12.8%)	11455 (11.3%)
Missing	7606 (8.1%)	931 (12.9%)	8537 (8.4%)
**total_component_score_subsequent_14**			
Did not Occur	59914 (63.5%)	3791 (52.4%)	63705 (62.8%)
Occurred	5499 (5.8%)	214 (3.0%)	5713 (5.6%)
Missing	28881 (30.6%)	3223 (44.6%)	32104 (31.6%)
**Impression_Diarrhoea/Constipation**			
Did not Occur	93762 (99.4%)	7211 (99.8%)	100973 (99.5%)
Occurred	532 (0.6%)	17 (0.2%)	549 (0.5%)
**Location_Public Place**			
Did not Occur	80597 (85.5%)	6052 (83.7%)	86649 (85.4%)
Occurred	2710 (2.9%)	335 (4.6%)	3045 (3.0%)
Missing	10987 (11.7%)	841 (11.6%)	11828 (11.7%)
**drug_Activated.Charcoal**			
Did not Occur	94145 (99.8%)	7219 (99.9%)	101364 (99.8%)
Occurred	149 (0.2%)	9 (0.1%)	158 (0.2%)
**immob_Other**			
Did not Occur	93931 (99.6%)	7225 (100.0%)	101156 (99.6%)
Occurred	363 (0.4%)	3 (0.0%)	366 (0.4%)
**total_component_score_primary_12**			
Did not Occur	90588 (96.1%)	6907 (95.6%)	97495 (96.0%)
Occurred	805 (0.9%)	50 (0.7%)	855 (0.8%)
Missing	2901 (3.1%)	271 (3.7%)	3172 (3.1%)
**Impression_Asthma**			
Did not Occur	93760 (99.4%)	7165 (99.1%)	100925 (99.4%)
Occurred	534 (0.6%)	63 (0.9%)	597 (0.6%)
**drug_Ondansetron**			
Did not Occur	91736 (97.3%)	7181 (99.3%)	98917 (97.4%)
Occurred	2558 (2.7%)	47 (0.7%)	2605 (2.6%)

**epr_news_score_5**			
Did not Occur	81516 (86.4%)	6111 (84.5%)	87627 (86.3%)
Occurred	5172 (5.5%)	186 (2.6%)	5358 (5.3%)
Missing	7606 (8.1%)	931 (12.9%)	8537 (8.4%)
**drug_Salbutamol**			
Did not Occur	89012 (94.4%)	7103 (98.3%)	96115 (94.7%)
Occurred	5282 (5.6%)	125 (1.7%)	5407 (5.3%)
**Impression_Cold & flu**			
Did not Occur	94186 (99.9%)	7207 (99.7%)	101393 (99.9%)
Occurred	108 (0.1%)	21 (0.3%)	129 (0.1%)
**psyc_CatastrophicHaemorrhage_No**			
Did not Occur	37 (0.0%)	4 (0.1%)	41 (0.0%)
Occurred	71580 (75.9%)	5529 (76.5%)	77109 (76.0%)
Missing	22677 (24.0%)	1695 (23.5%)	24372 (24.0%)
**Impression_Hypertension**			
Did not Occur	93941 (99.6%)	7187 (99.4%)	101128 (99.6%)
Occurred	353 (0.4%)	41 (0.6%)	394 (0.4%)
**Impression_Bite/sting**			
Did not Occur	94263 (100.0%)	7217 (99.8%)	101480 (100.0%)
Occurred	31 (0.0%)	11 (0.2%)	42 (0.0%)
**Impression_Poisoning**			
Did not Occur	94040 (99.7%)	7197 (99.6%)	101237 (99.7%)
Occurred	254 (0.3%)	31 (0.4%)	285 (0.3%)
