# Supplementary material for: The Safety INdEx of Prehospital On Scene Triage (SINEPOST) study: The development and validation of a risk prediction model to support ambulance clinical transport decisions on-scene
Source: PLoS One. 2022 Nov 16;17(11):e0276515. doi: 10.1371/journal.pone.0276515 (PMC9668173; doi:10.1371/journal.pone.0276515)
Supplement: S4 Appendix — (PDF) [file pone.0276515.s004.pdf]

Appendix S4: Hyperparameter values per cluster

Model	eta	max_depth	min_child _weight	subsample	Colsample _bytree	gamma	alpha	scale_pos _weight	n_rounds
Airedale	0.06	4	4	0.9	0.6	0.5	0.6	1	408
Barnsley	0.06	3	4	1	0.6	1	0.7	0.67	630
Bradford	0.06	3	2	0.7	0.9	0.5	0.6	2.1	395
Calderdale	0.06	3	2	0.9	0.6	0.5	0.8	0.68	477
Dewsbury	0.06	3	4	0.9	0.6	1	0.6	1.49	463
Doncaster	0.06	3	2	0.9	0.6	1	0.7	1.01	453
Harrogate	0.08	3	4	0.9	0.6	0	0.7	0.85	467
Huddersfield	0.06	3	2	0.9	0.9	0	0.7	0.83	516
Hull	0.06	3	4	0.9	0.6	1	0.7	0.9	472
Middlesborough	0.08	4	2	0.9	0.9	0.5	0.8	1.37	261
Leeds 1	0.06	3	4	0.9	0.6	0	0.7	0.68	485
Sheffield	0.06	3	4	0.9	0.6	0	0.6	1.13	411
Wakefield	0.06	4	2	0.7	0.6	1	0.7	1.06	330
Rotherham	0.06	3	4	0.9	0.6	0.5	0.8	0.81	524
Scarborough	0.06	3	4	0.9	0.9	0.5	0.6	0.41	577
Leeds 2	0.06	4	4	0.9	0.9	0	0.7	1.26	348
York	0.06	4	4	0.9	0.9	1	0.8	0.86	367
