# Supplementary material for: The Safety INdEx of Prehospital On Scene Triage (SINEPOST) study: The development and validation of a risk prediction model to support ambulance clinical transport decisions on-scene
Source: PLoS One. 2022 Nov 16;17(11):e0276515. doi: 10.1371/journal.pone.0276515 (PMC9668173; doi:10.1371/journal.pone.0276515)
Supplement: S5 Appendix — (PDF) [file pone.0276515.s005.pdf]

Appendix S5: ROC and calibration curves for the IECVmodels

Airedale

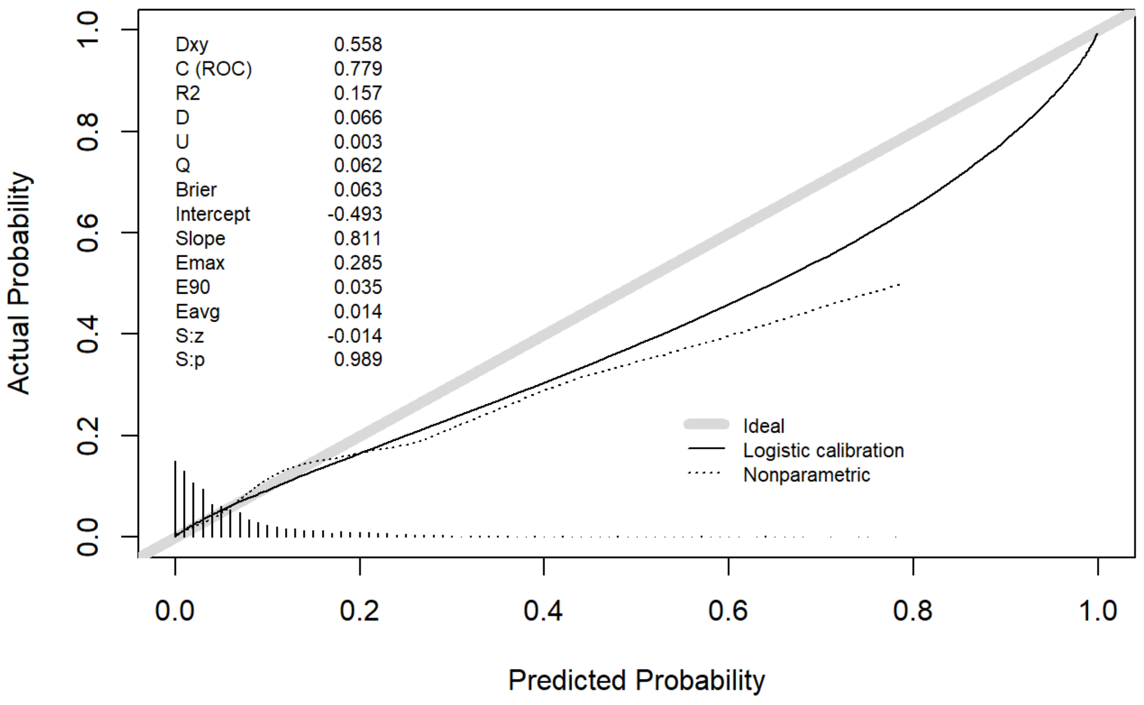

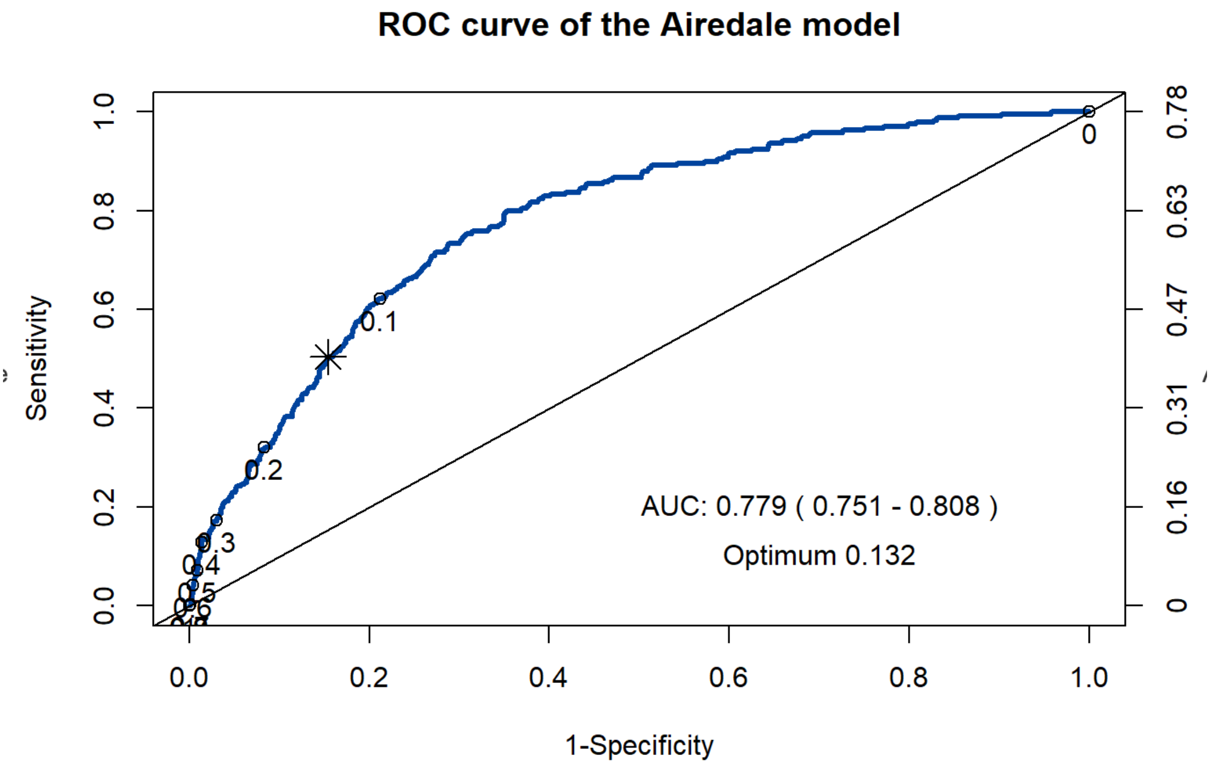

Barnsley

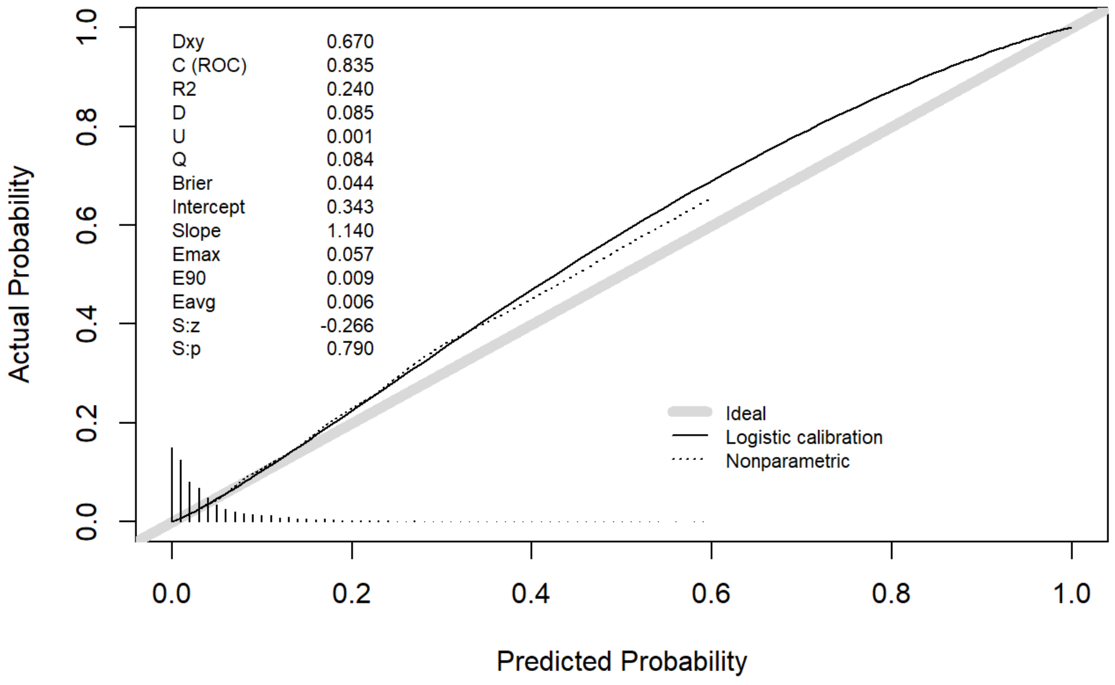
Dxy 0.670
C (ROC) 0.835
R2 0.240
D 0.085
U 0.001
Q 0.084
Brier 0.044
Intercept 0.343
Slope 1.140
Emax 0.057
E90 0.009
Eavg 0.006
S:z -0.266
S:p 0.790
Ideal
Logistic calibration
Nonparametric
Actual Probability
Predicted Probability
0.0
0.2
0.4
0.6
0.8
1.0

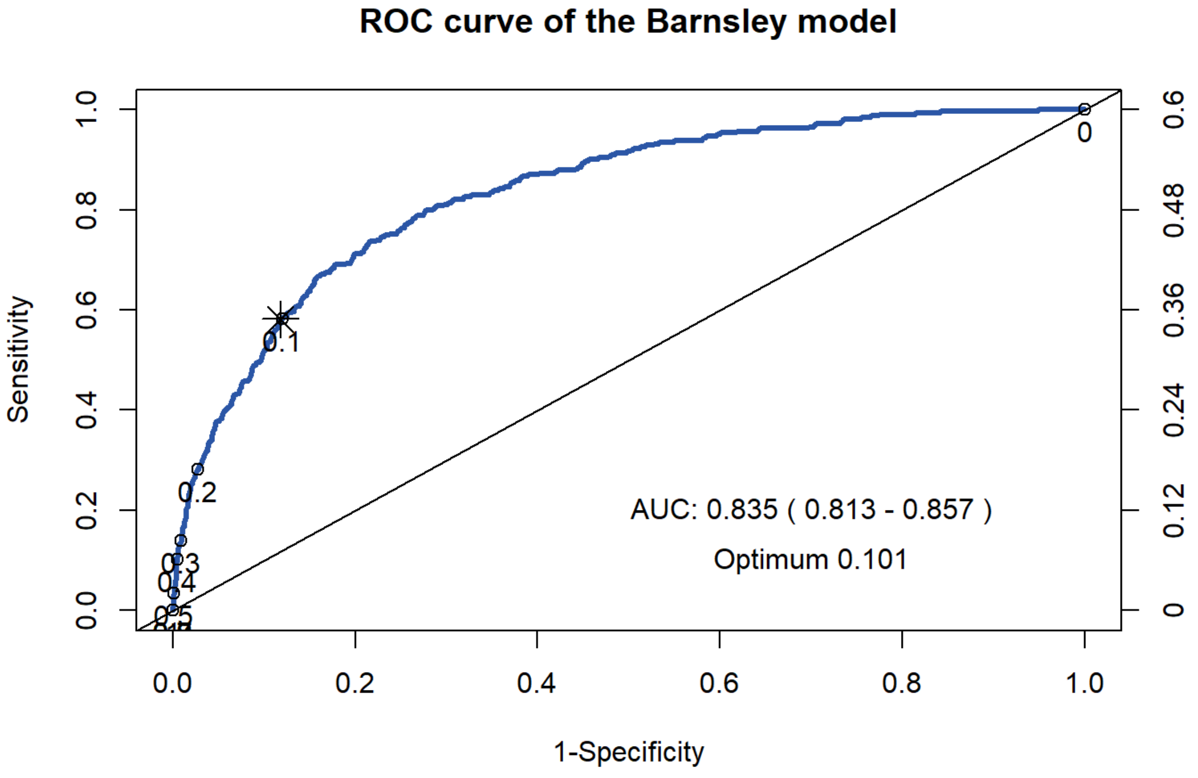
ROC curve of the Barnsley model
Sensitivity
1-Specificity
AUC: 0.835 (0.813 - 0.857)
Optimum 0.101
0.1
0.2
0
0.0
0.2
0.4
0.6
0.8
1.0
0
0.12
0.24
0.36
0.48
0.6

Bradford

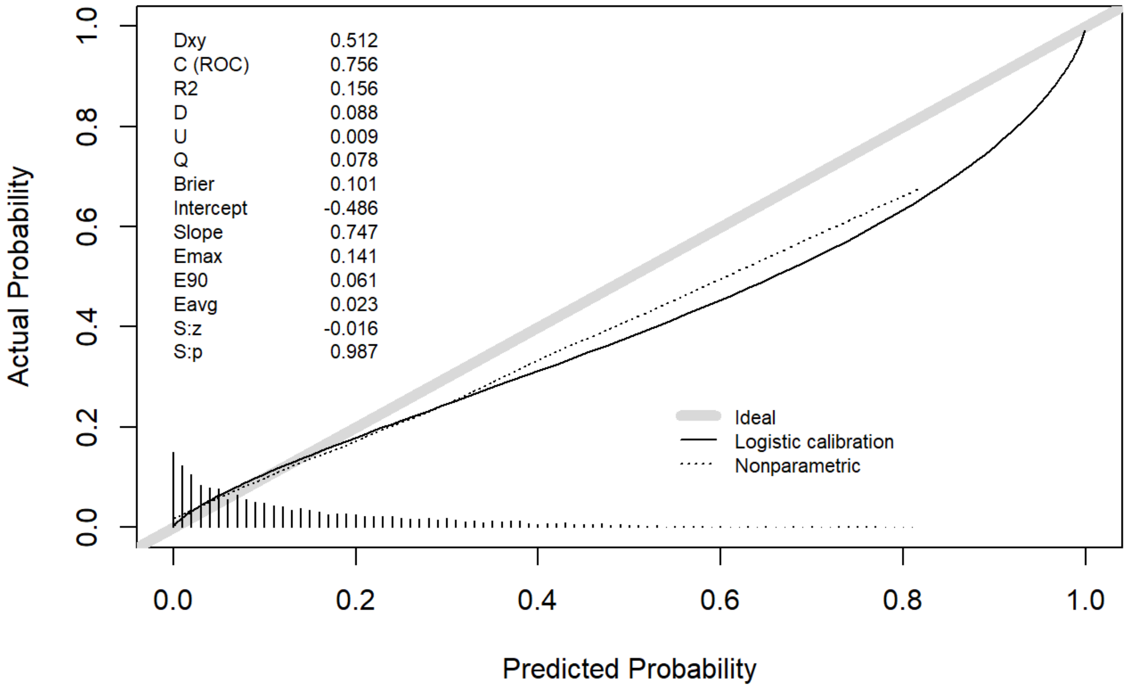

Dxy 0.512
C (ROC) 0.756
R2 0.156
D 0.088
U 0.009
Q 0.078
Brier 0.101
Intercept -0.486
Slope 0.747
Emax 0.141
E90 0.061
Eavg 0.023
S:z -0.016
S:p 0.987
Ideal
Logistic calibration
Nonparametric
Actual Probability
Predicted Probability

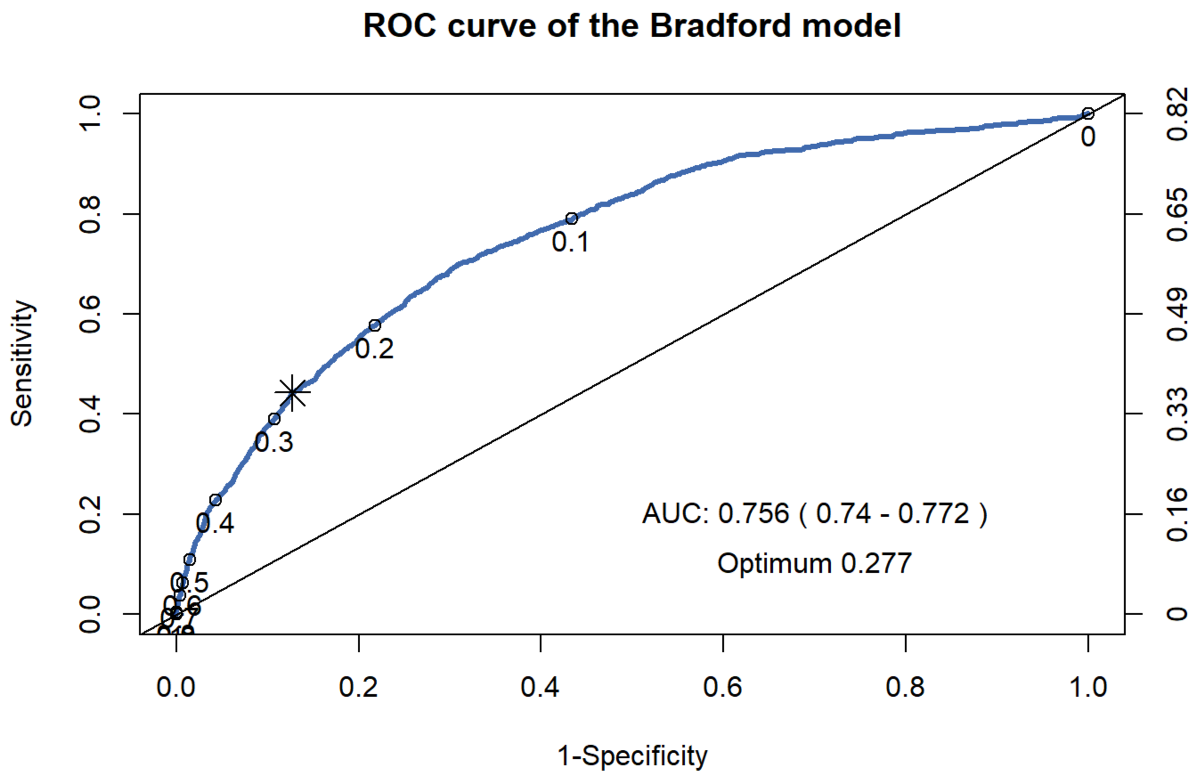

ROC curve of the Bradford model
AUC: 0.756 (0.74 - 0.772)
Optimum 0.277
Sensitivity
1-Specificity

Calderdale

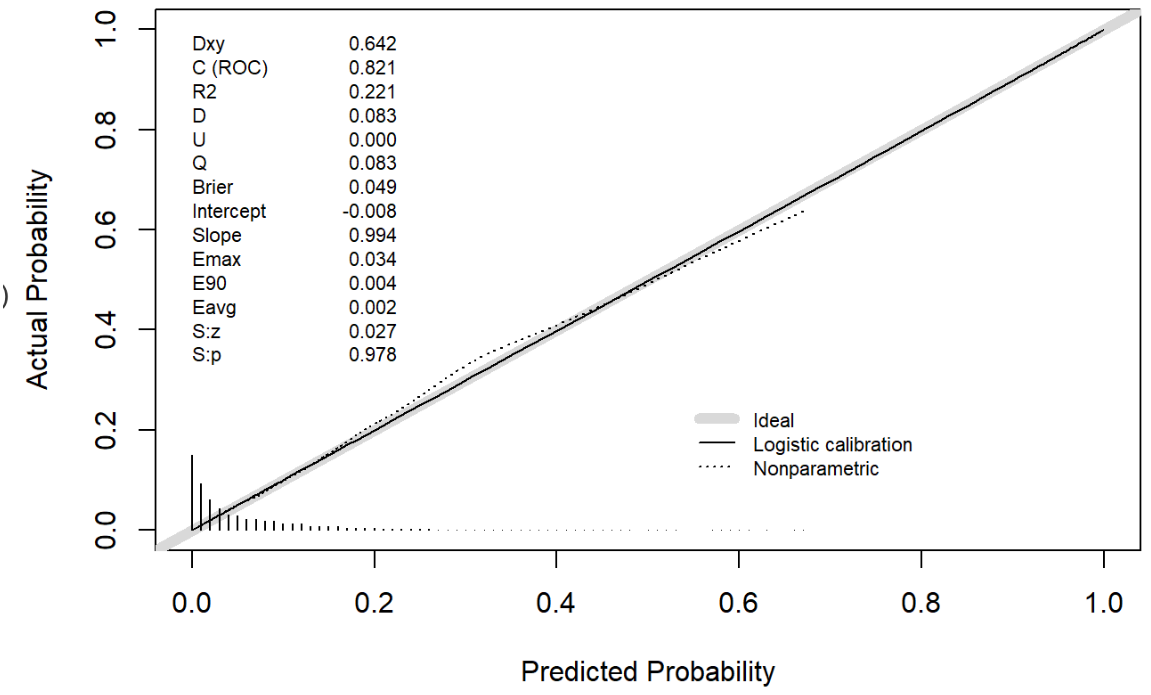

Dxy 0.642
C (ROC) 0.821
R2 0.221
D 0.083
U 0.000
Q 0.083
Brier 0.049
Intercept -0.008
Slope 0.994
Emax 0.034
E90 0.004
Eavg 0.002
S:z 0.027
S:p 0.978
Ideal
Logistic calibration
Nonparametric
Actual Probability
Predicted Probability
0.0
0.2
0.4
0.6
0.8
1.0

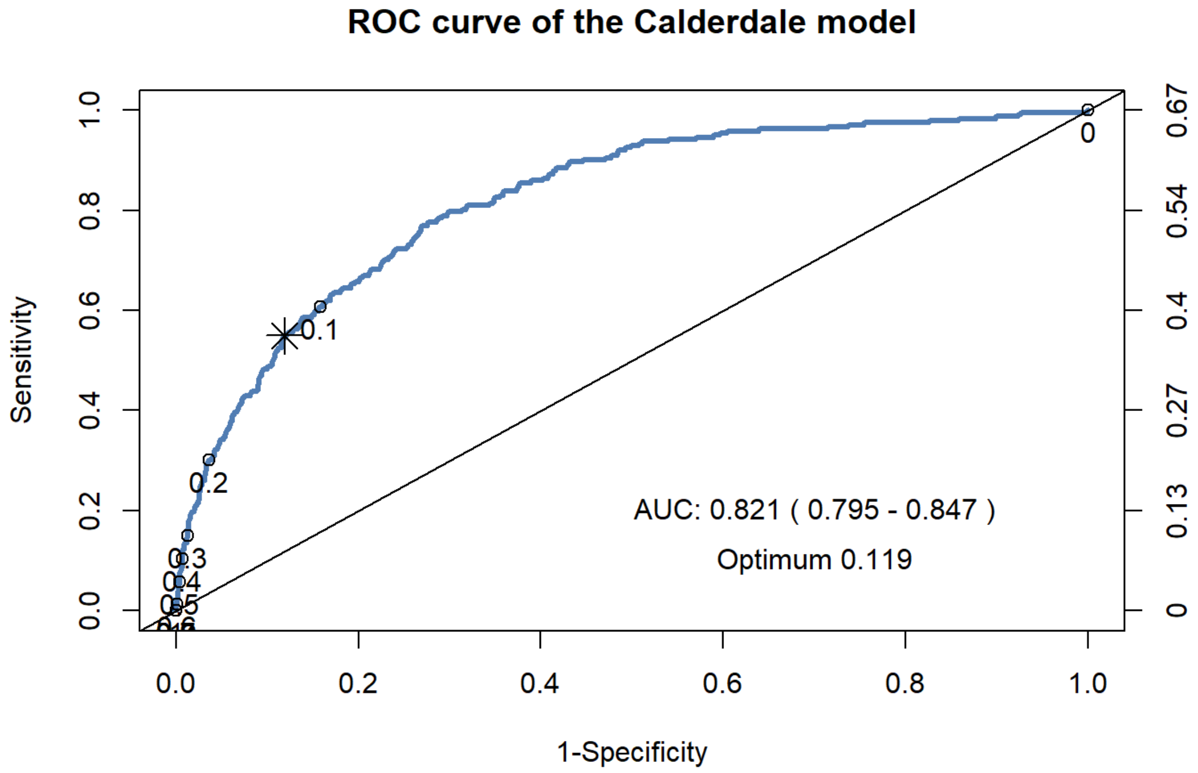

ROC curve of the Calderdale model
AUC: 0.821 (0.795 - 0.847)
Optimum 0.119
Sensitivity
1-Specificity
0.0
0.2
0.4
0.6
0.8
1.0
0
0.13
0.27
0.4
0.54
0.67
0.1
0.2
0.3
0.4
0.5

Dewsbury

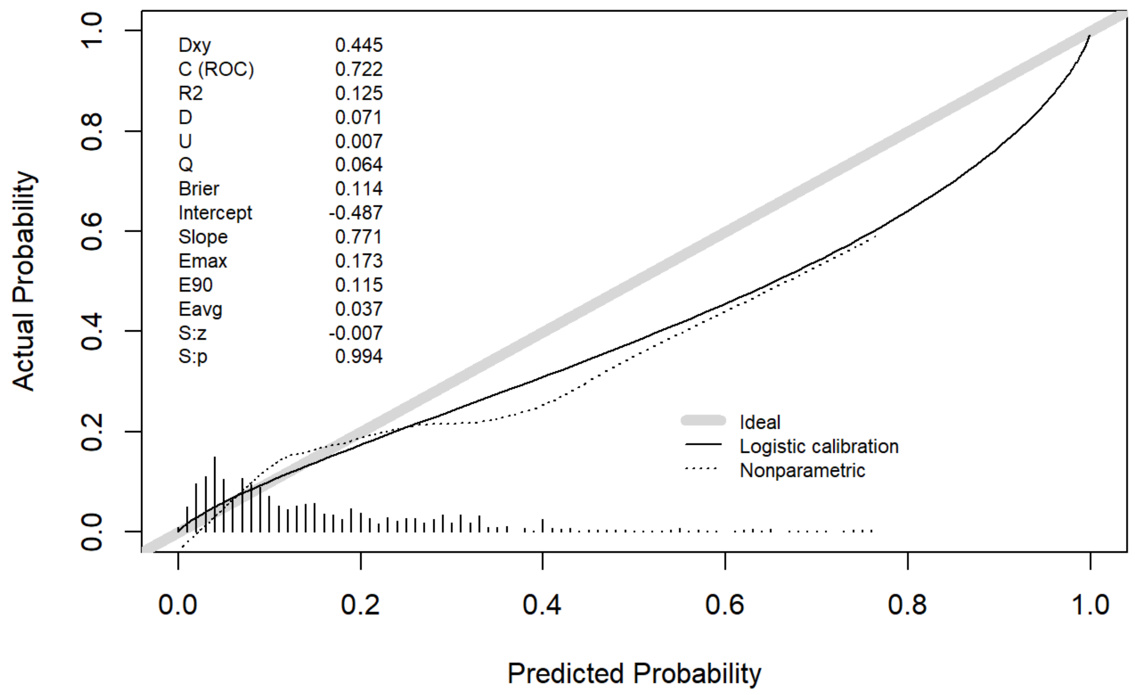

ROC curve of the Dewsbury model

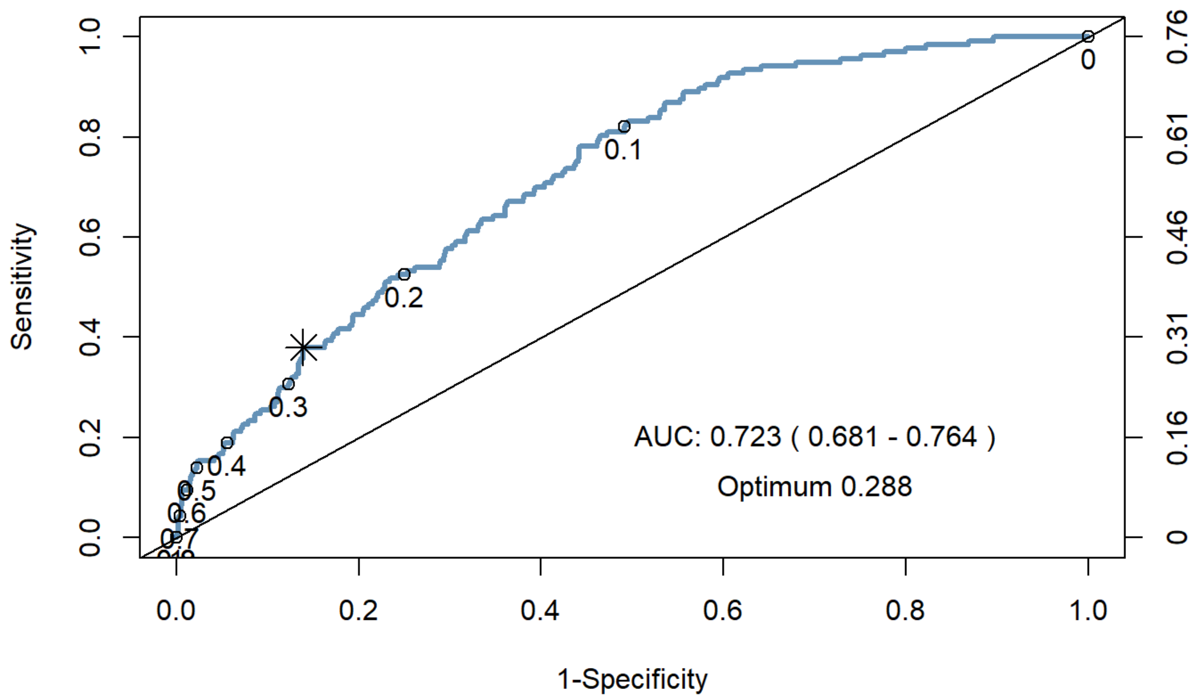

Doncaster

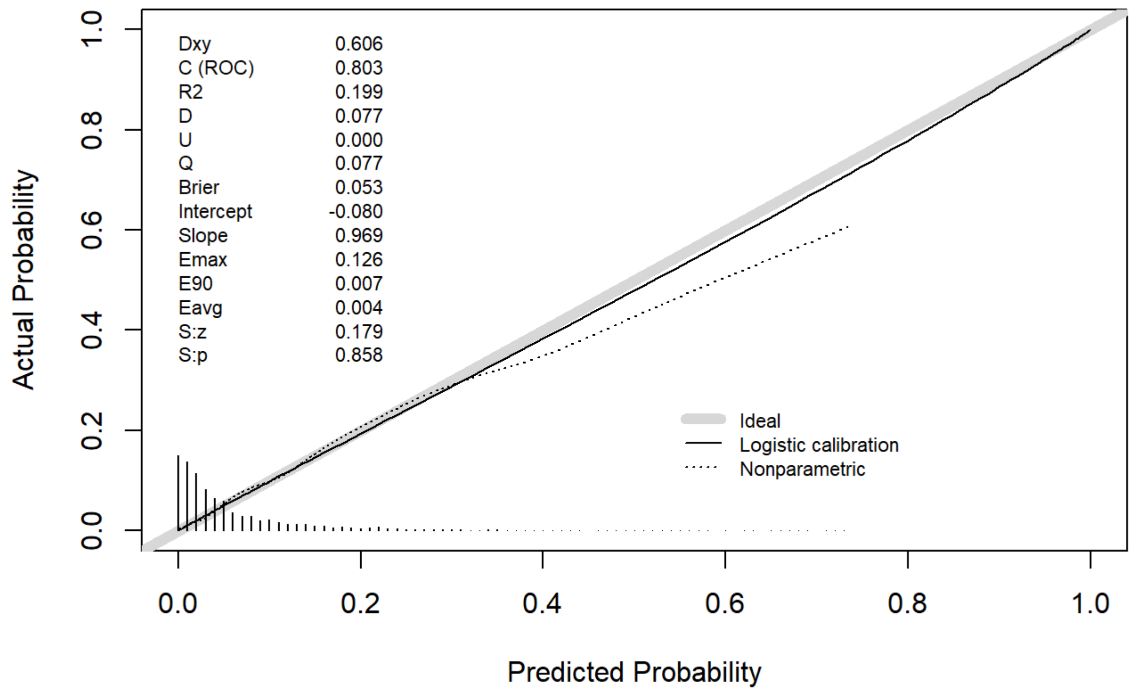

ROC curve of the Doncaster model

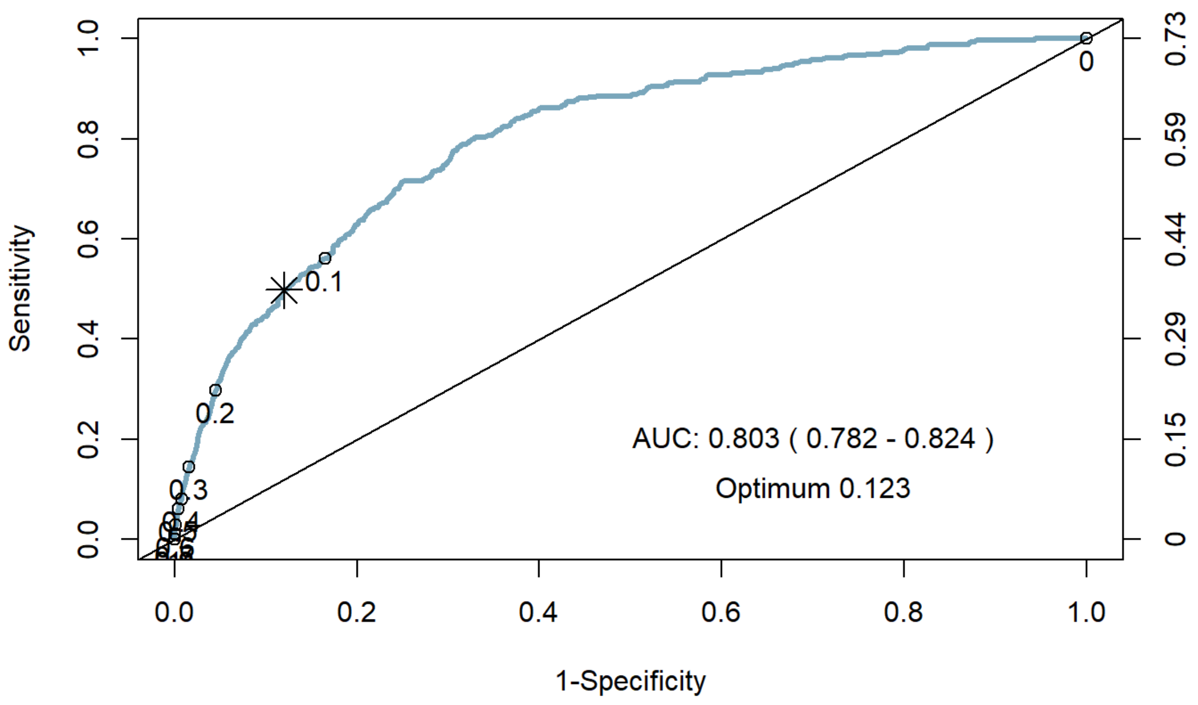

Harrogate

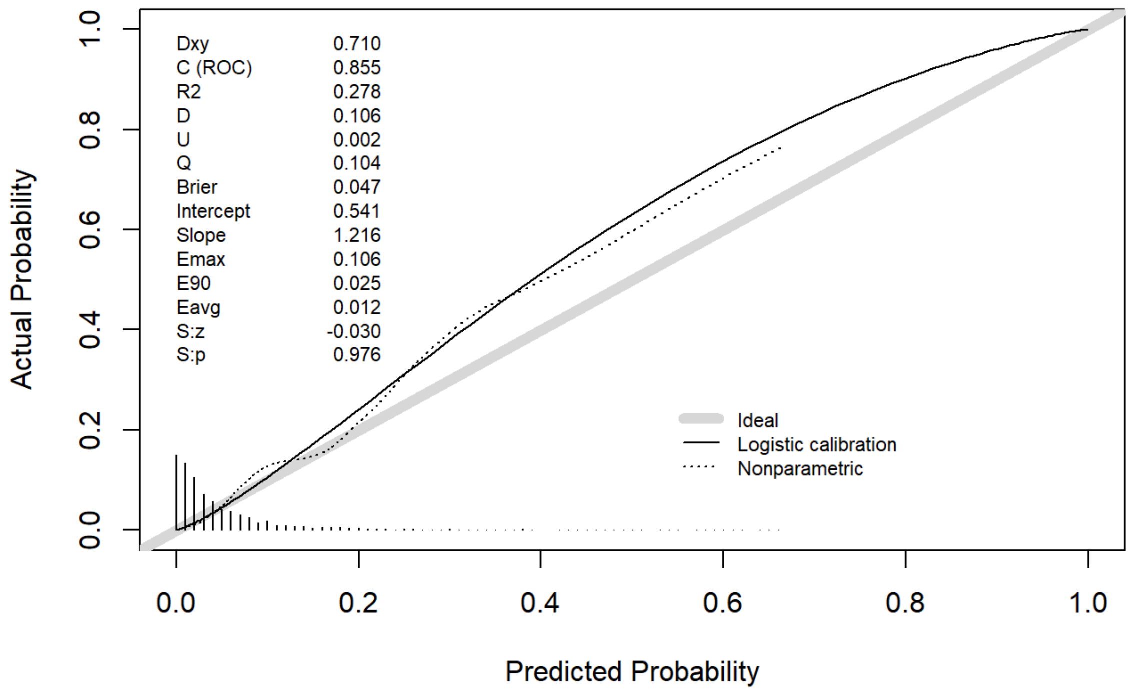
Dxy 0.710
C (ROC) 0.855
R2 0.278
D 0.106
U 0.002
Q 0.104
Brier 0.047
Intercept 0.541
Slope 1.216
Emax 0.106
E90 0.025
Eavg 0.012
S:z -0.030
S:p 0.976
Ideal
Logistic calibration
Nonparametric
Actual Probability
Predicted Probability
0.0
0.2
0.4
0.6
0.8
1.0

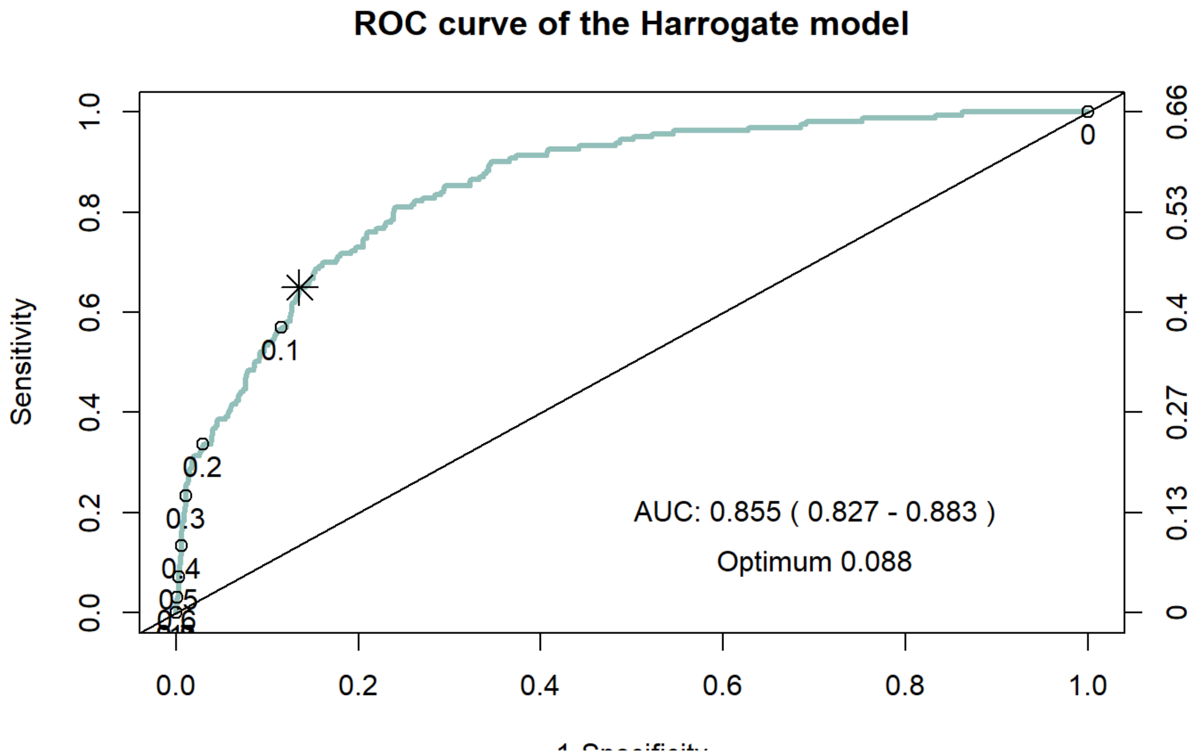
ROC curve of the Harrogate model
AUC: 0.855 (0.827 - 0.883)
Optimum 0.088
Sensitivity
0.0
0.2
0.4
0.6
0.8
1.0
0
0.13
0.27
0.4
0.53
0.66
0.1
0.2
0.3
0.4
0.5

Huddersfield

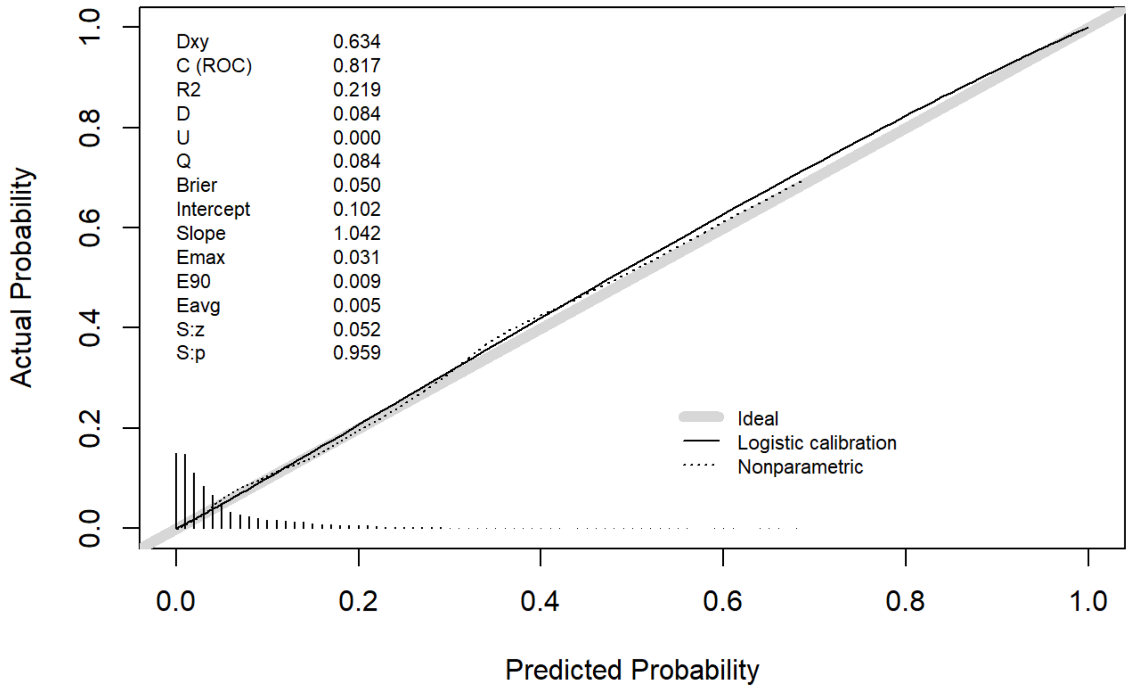

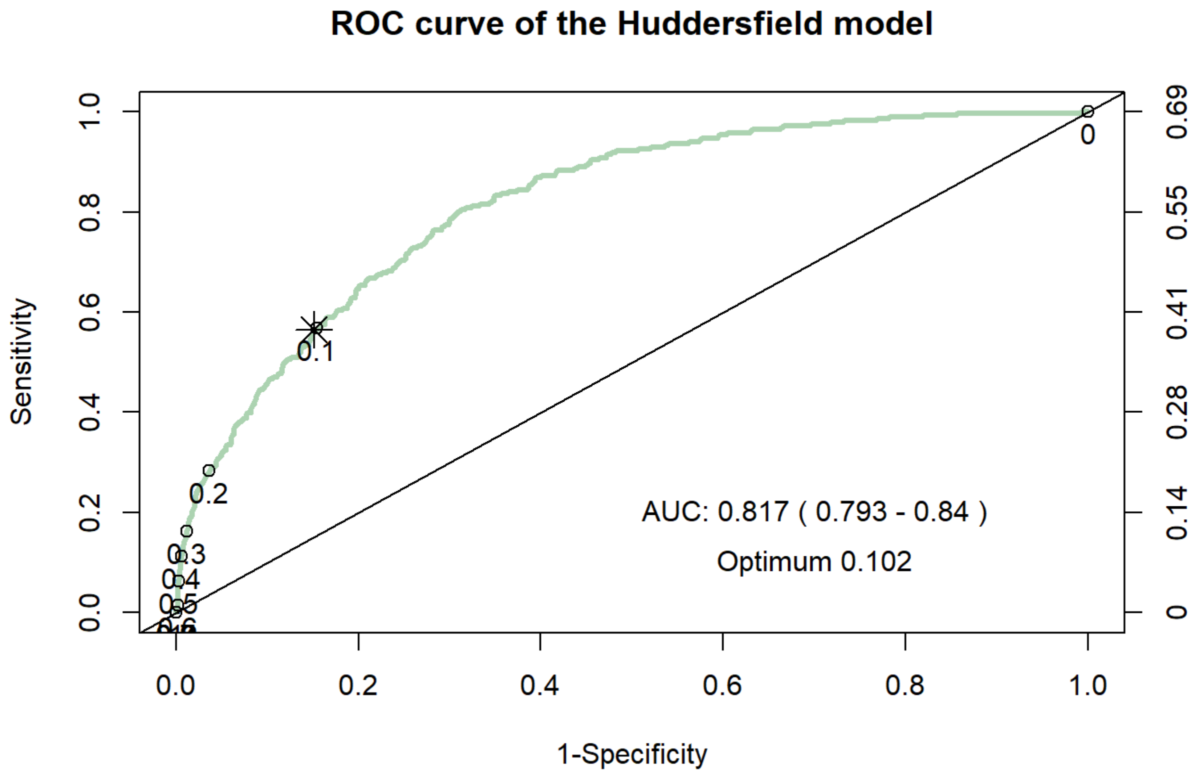

Hull

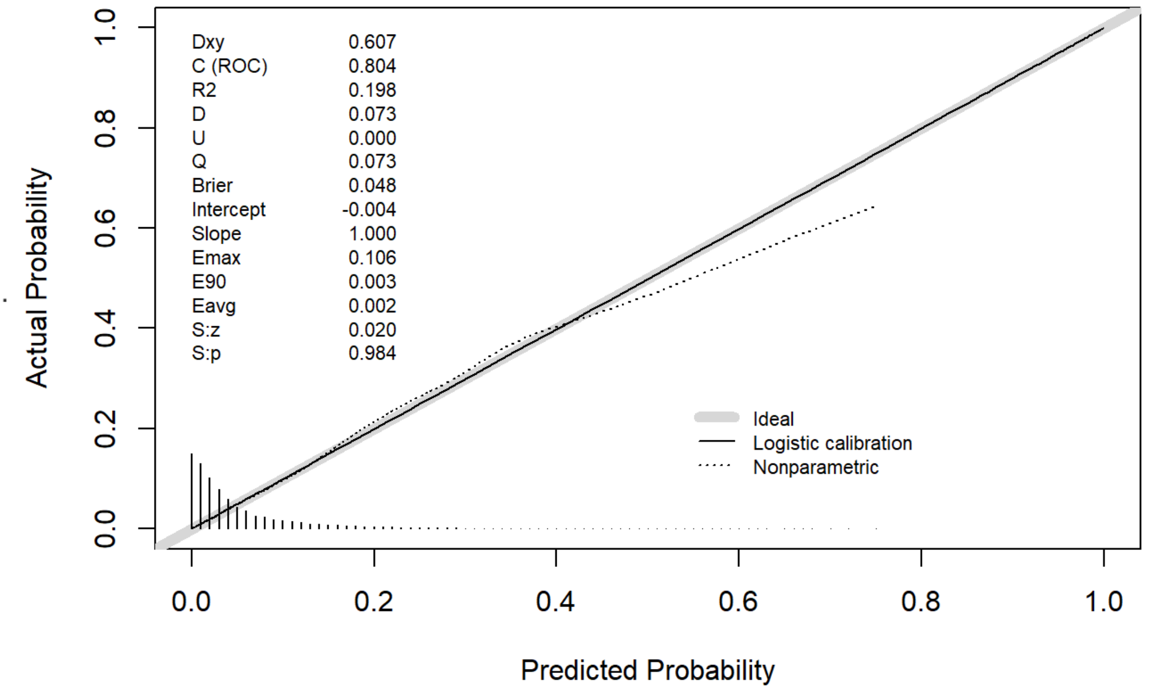

Dxy 0.607
C (ROC) 0.804
R2 0.198
D 0.073
U 0.000
Q 0.073
Brier 0.048
Intercept -0.004
Slope 1.000
Emax 0.106
E90 0.003
Eavg 0.002
S:z 0.020
S:p 0.984
Ideal
Logistic calibration
Nonparametric
Actual Probability
Predicted Probability

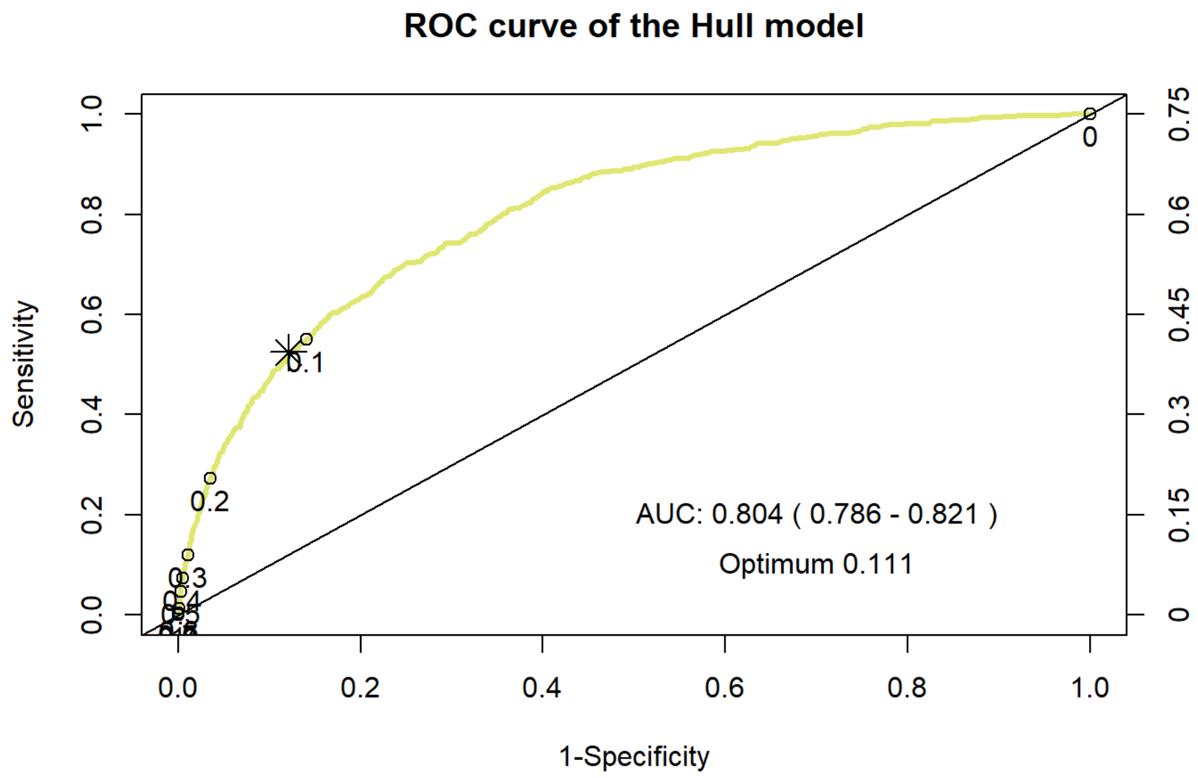

ROC curve of the Hull model
AUC: 0.804 (0.786 - 0.821)
Optimum 0.111
Sensitivity
1-Specificity

James Cook University Hospital Middlesbrough

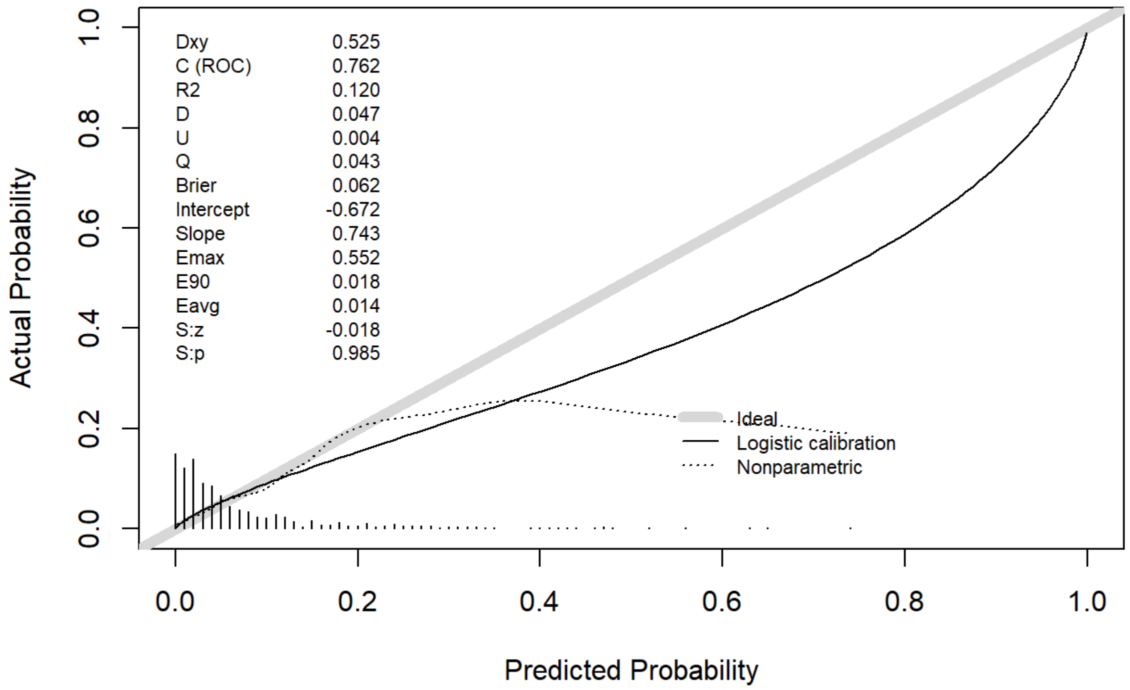

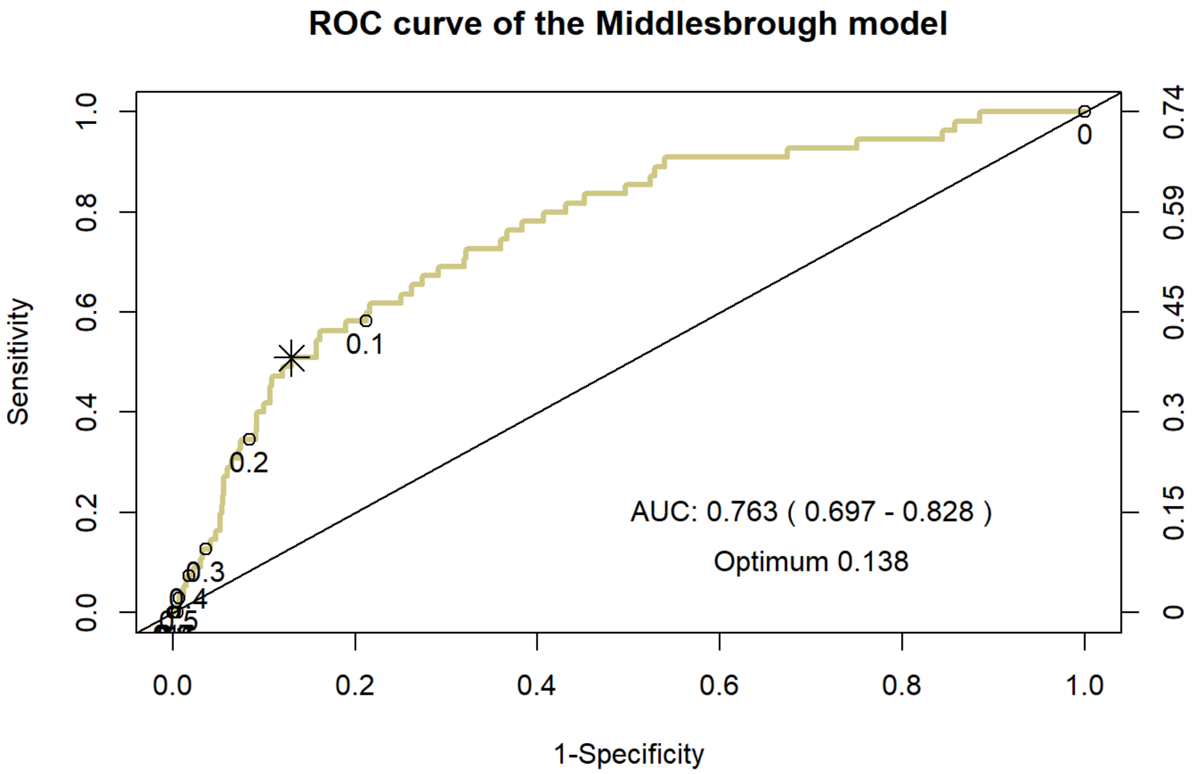

Leeds General Infirmary (LGI)

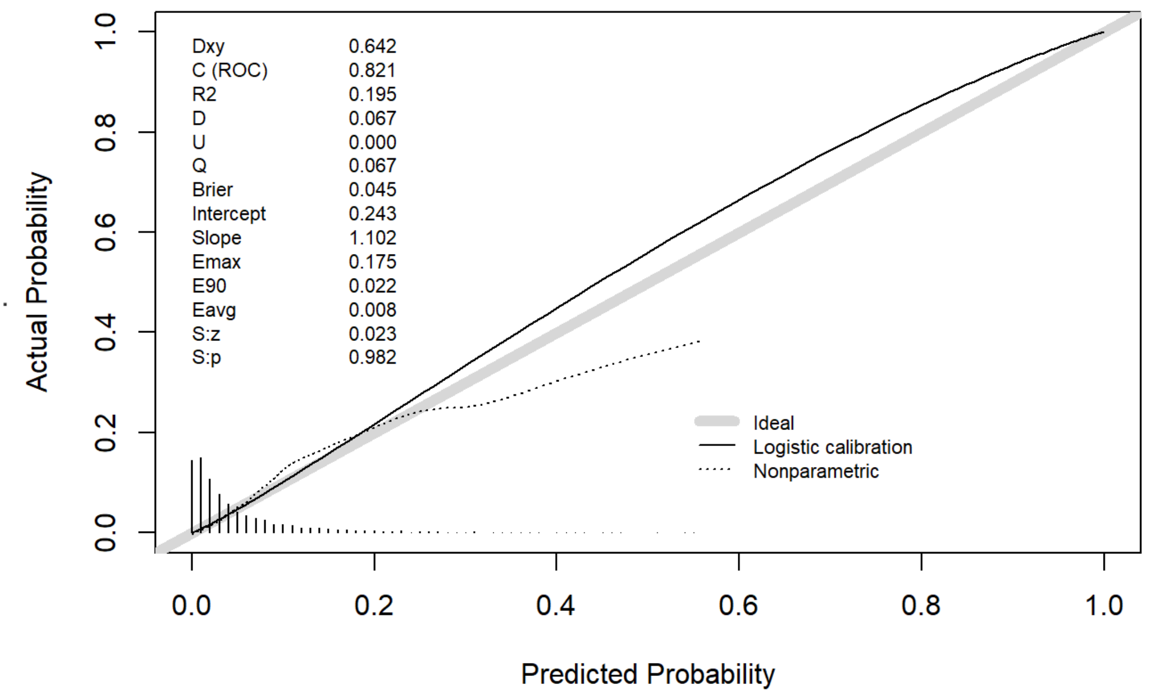

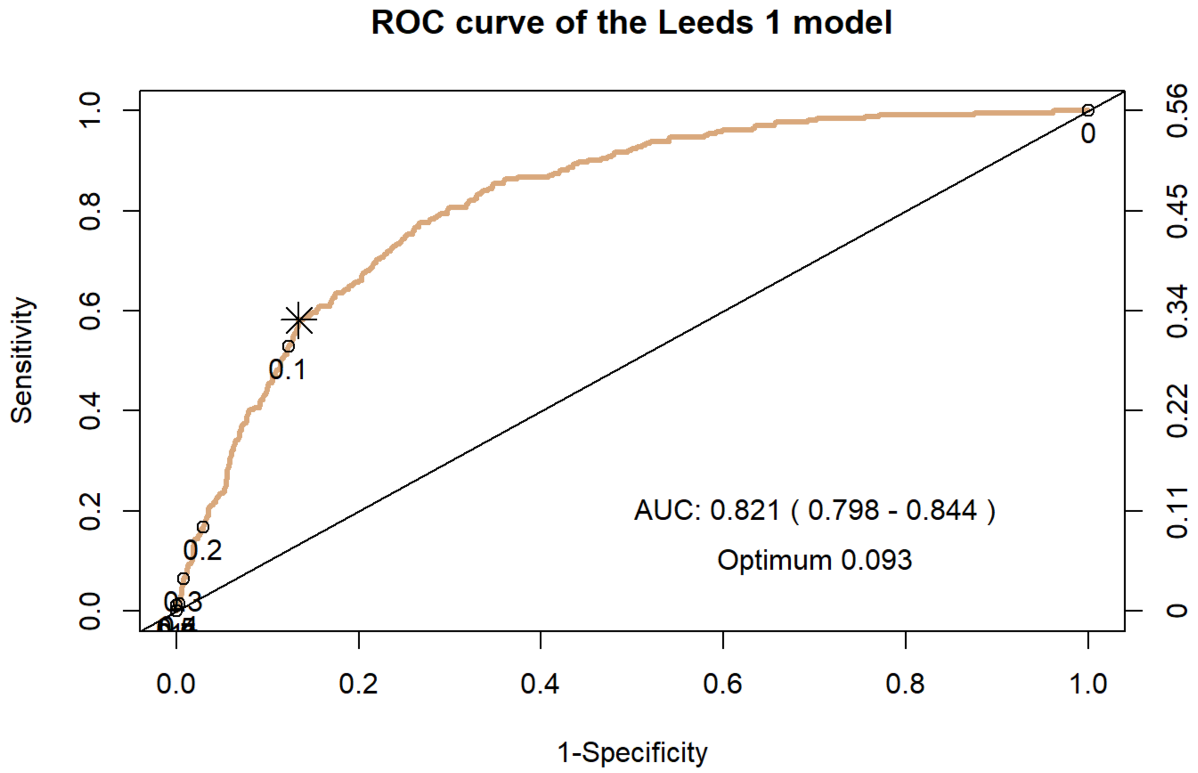

Northern General Hospital Sheffield

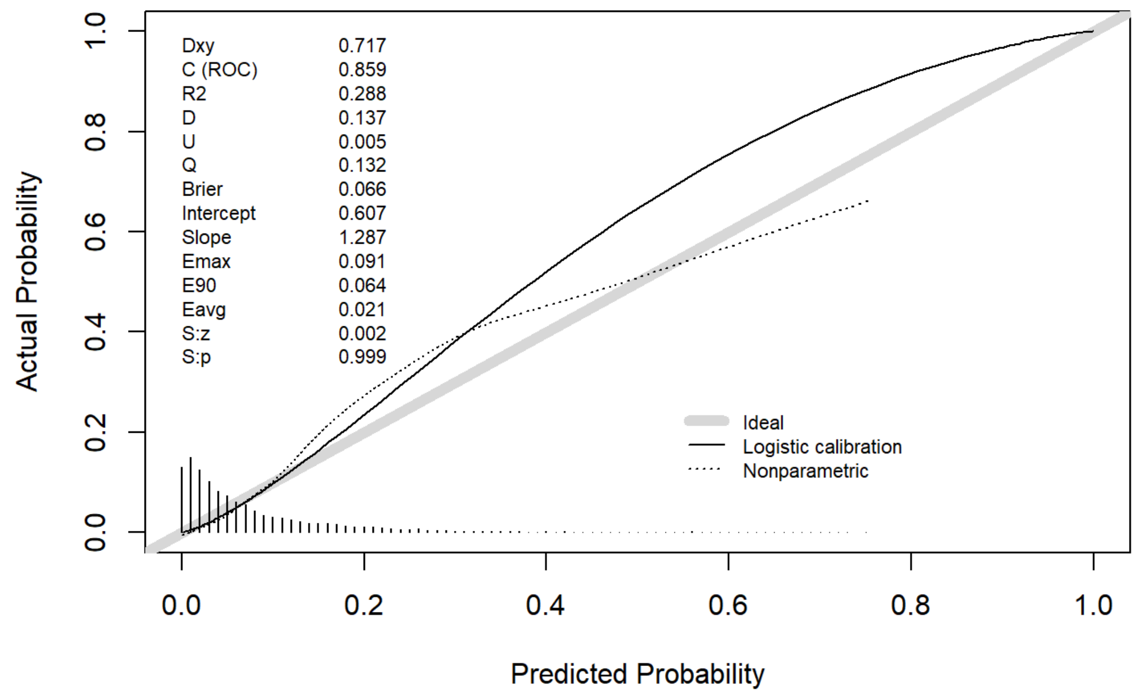

ROC curve of the Sheffield model

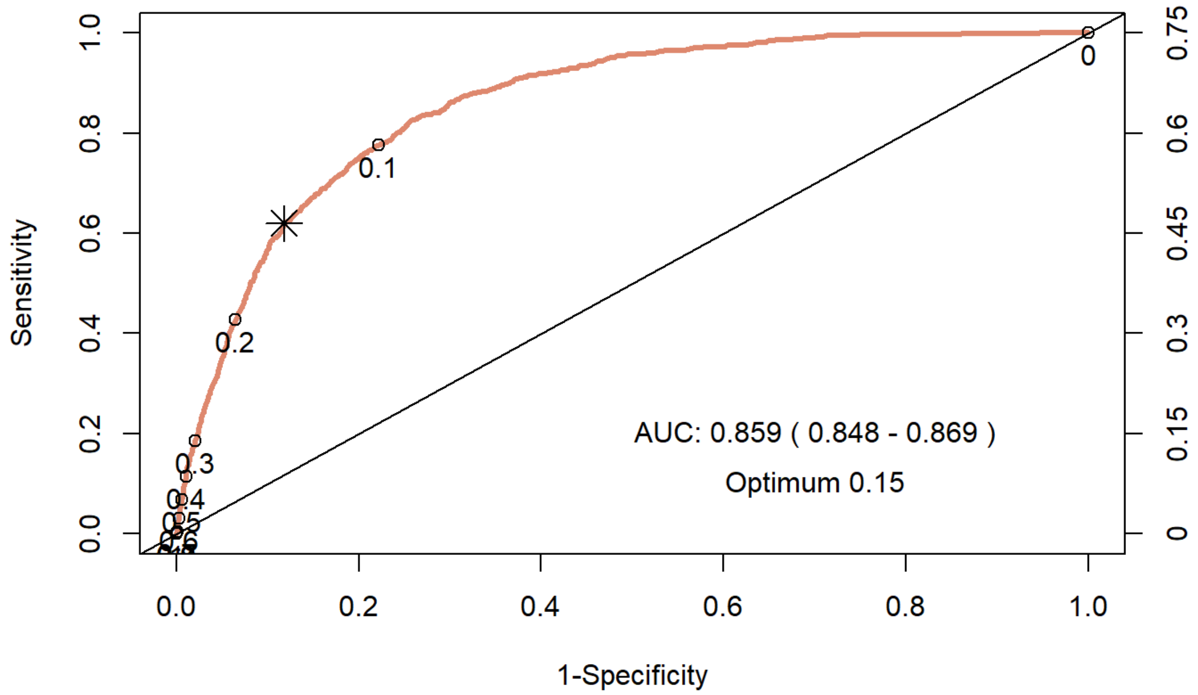

Pinderfields Hospital Wakefield

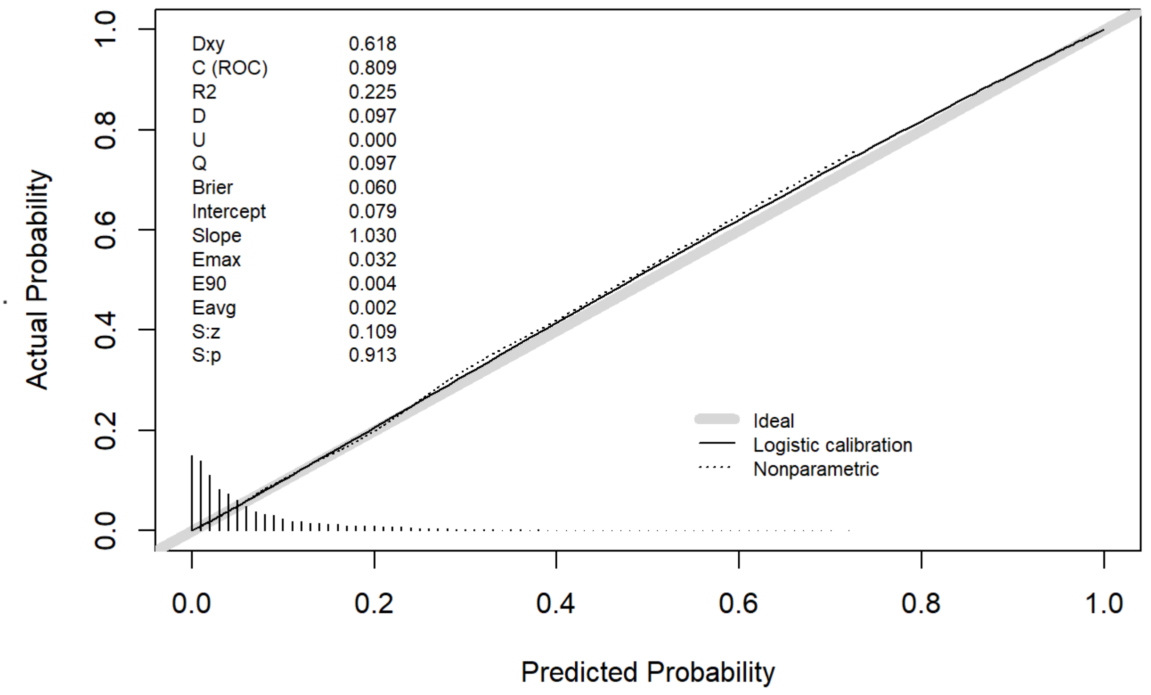

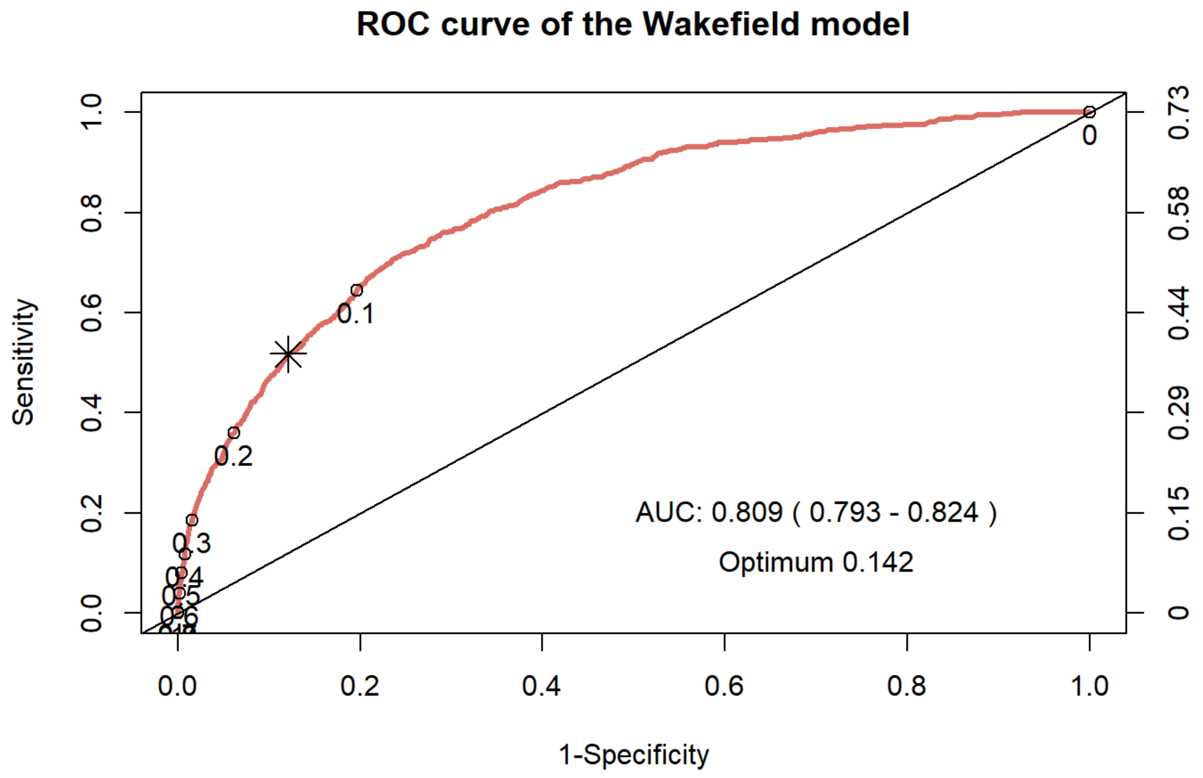

Rotherham

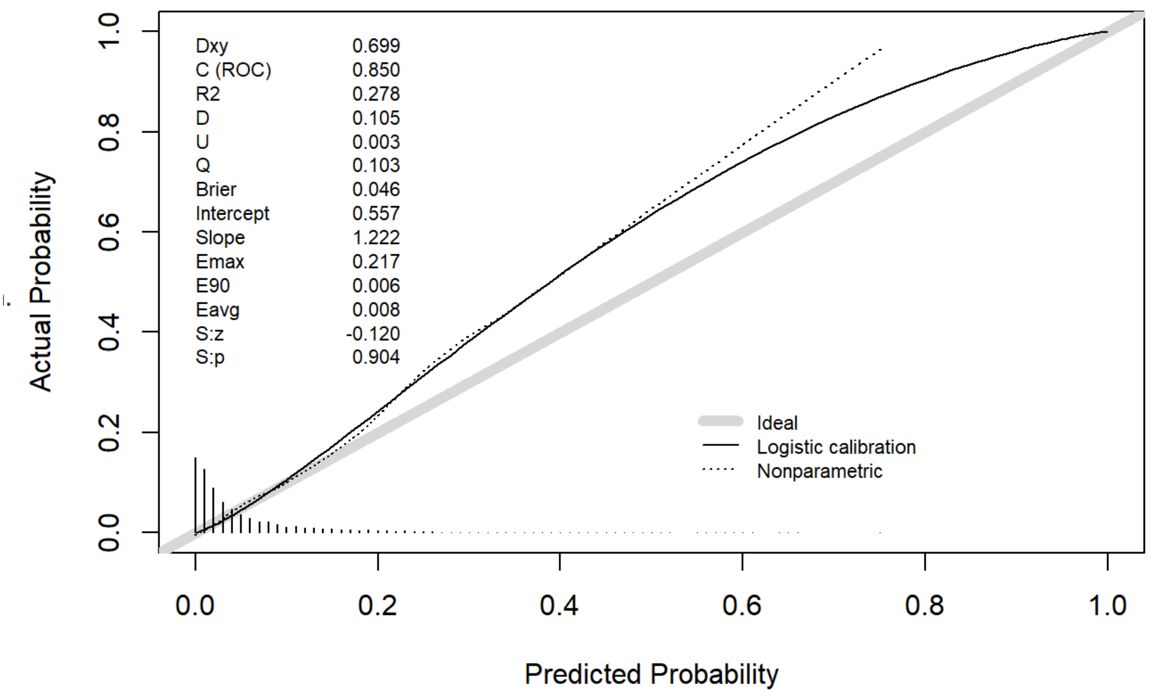

Dxy 0.699
C (ROC) 0.850
R2 0.278
D 0.105
U 0.003
Q 0.103
Brier 0.046
Intercept 0.557
Slope 1.222
Emax 0.217
E90 0.006
Eavg 0.008
S:z -0.120
S:p 0.904
Ideal
Logistic calibration
Nonparametric
Actual Probability
Predicted Probability

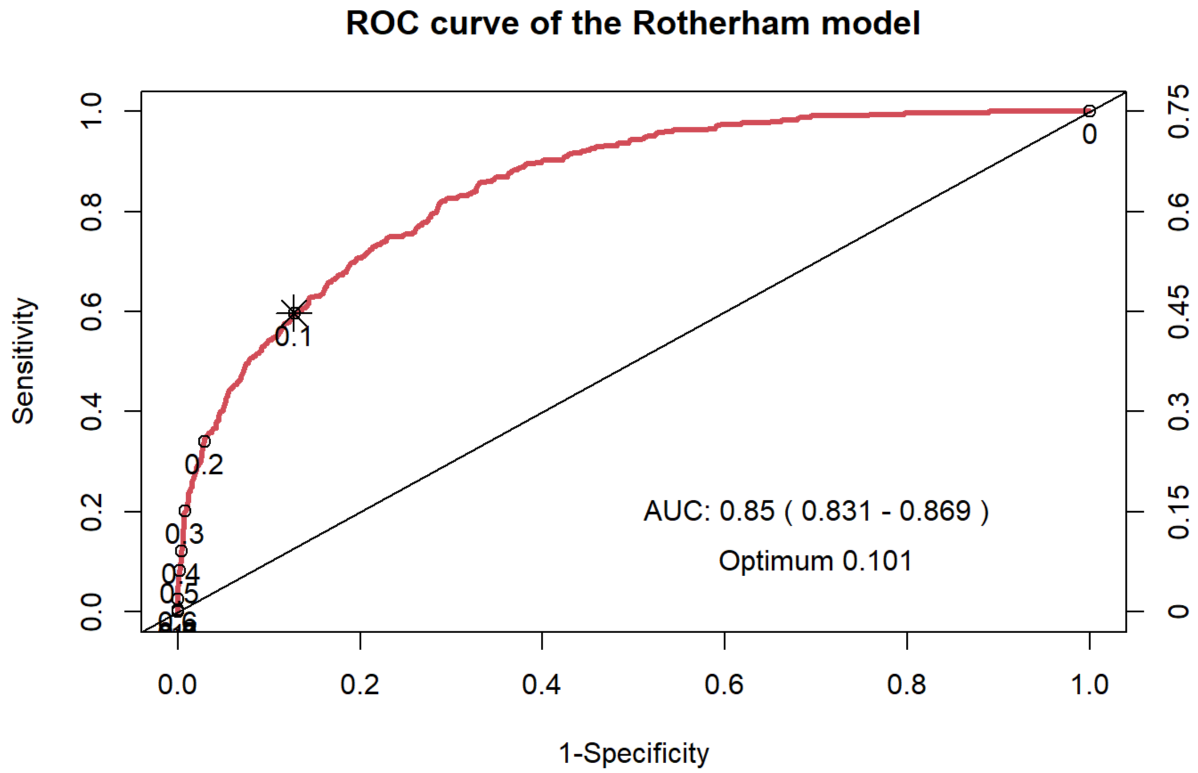

ROC curve of the Rotherham model
AUC: 0.85 (0.831 - 0.869)
Optimum 0.101
Sensitivity
1-Specificity

Scarborough

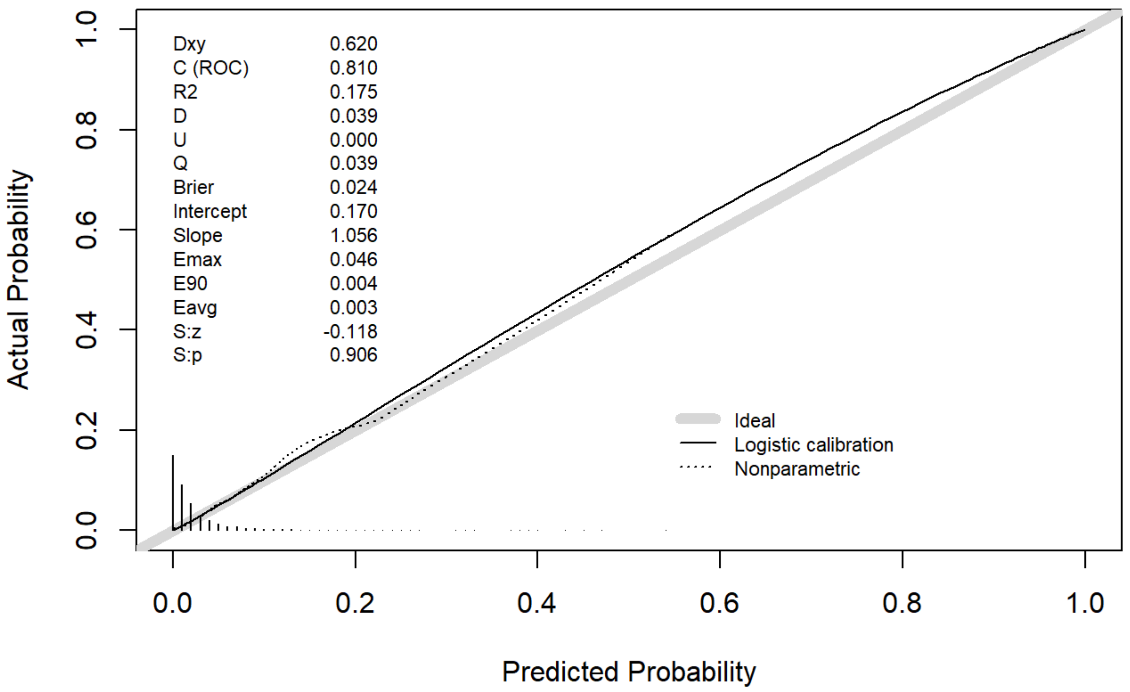

ROC curve of the Scarborough model

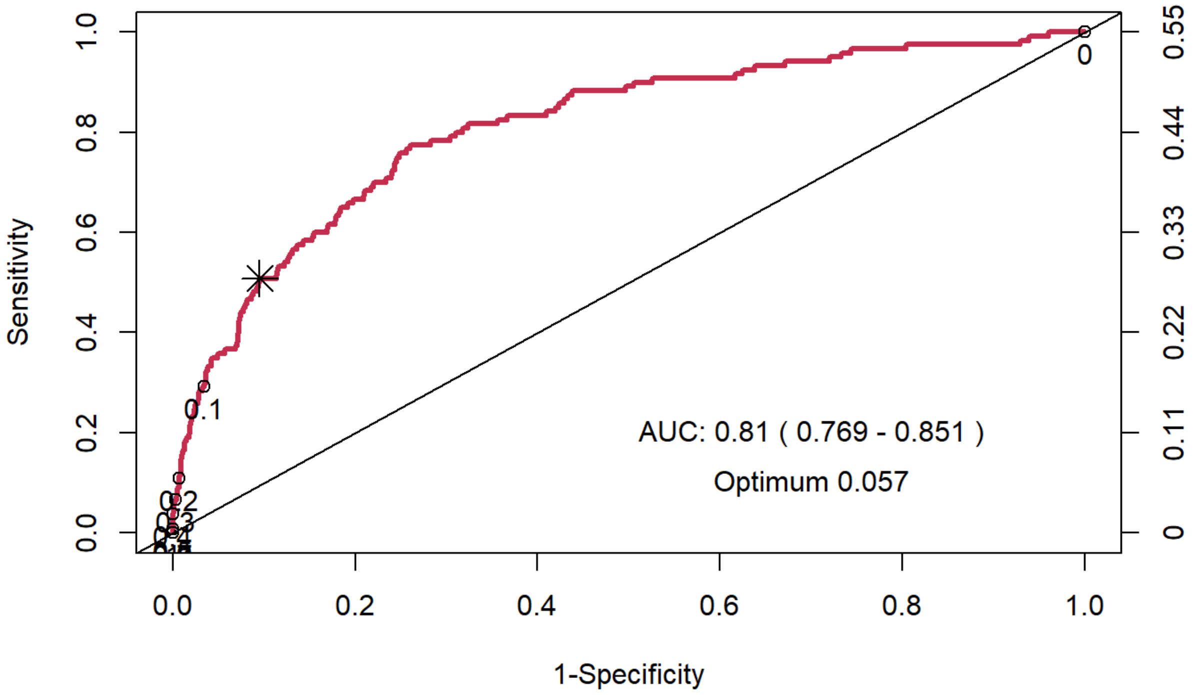

St. James's Hospital Leeds

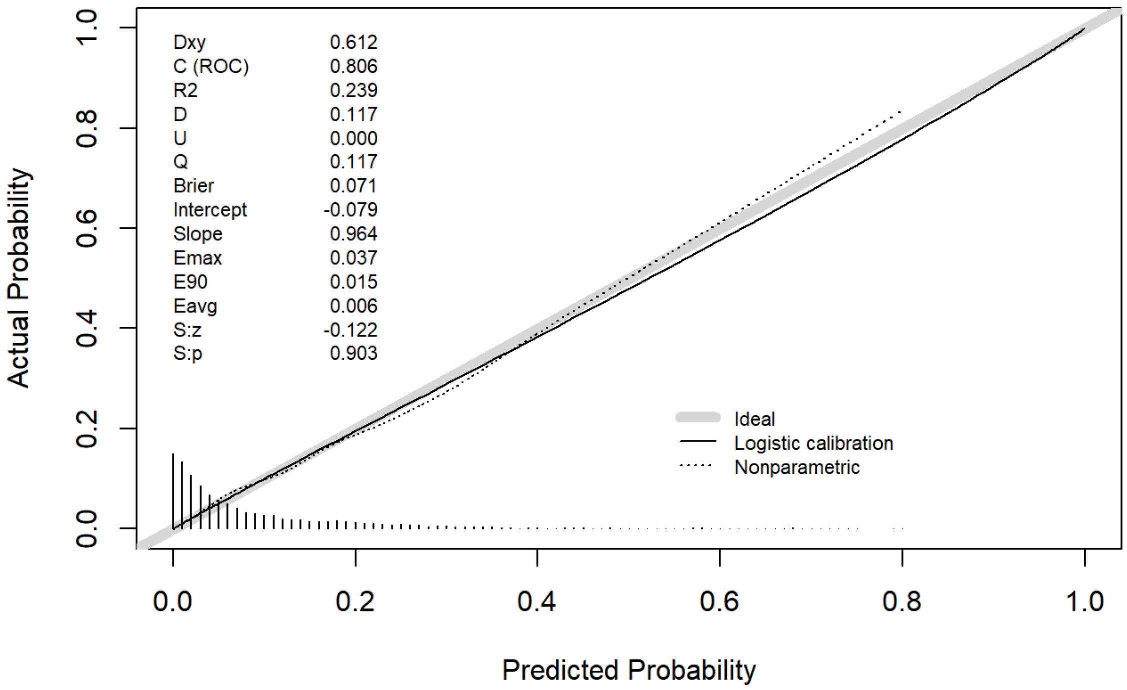

ROC curve of the Leeds 2 model

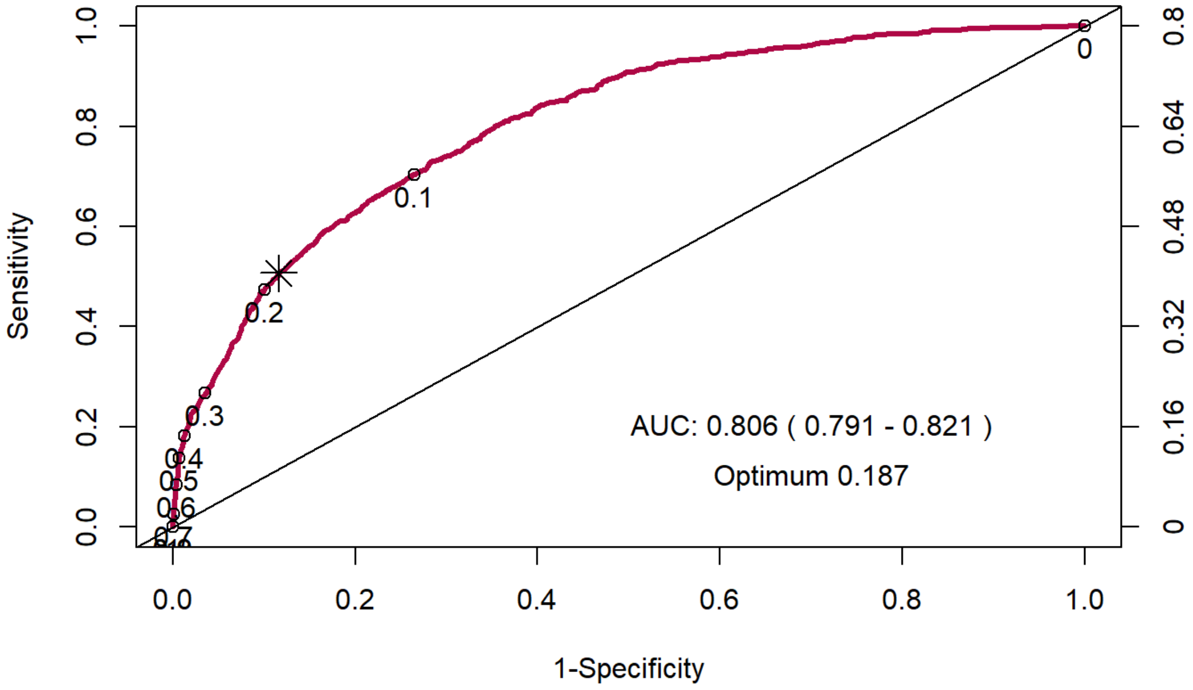

York

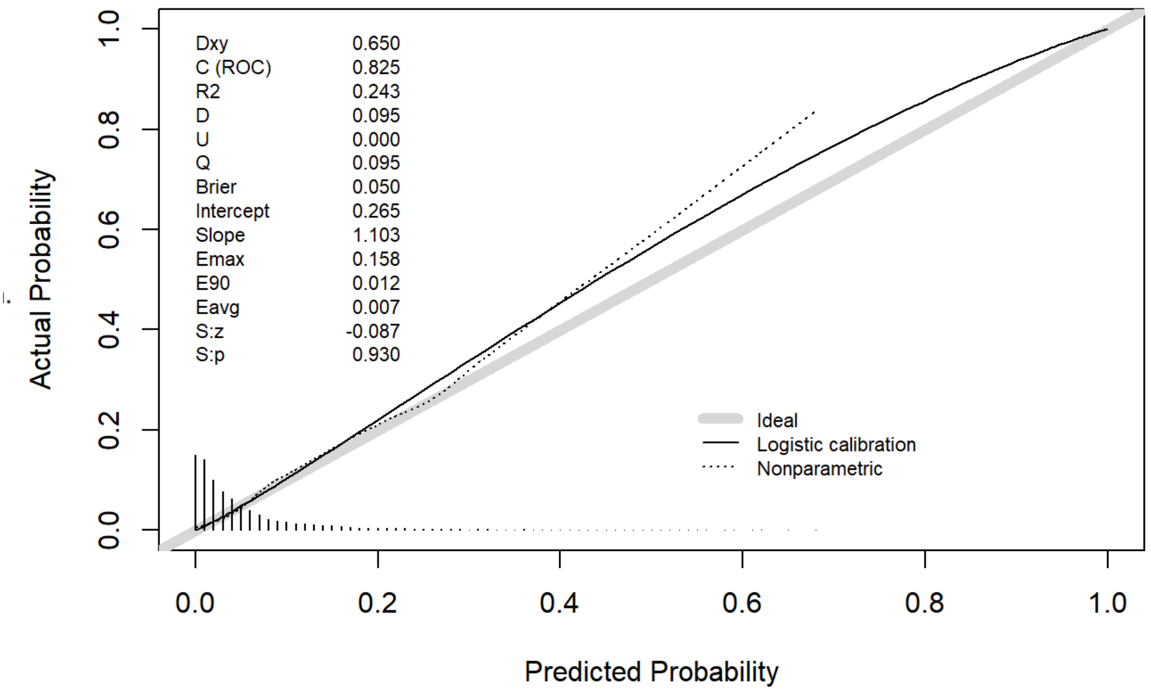
Dxy 0.650
C (ROC) 0.825
R2 0.243
D 0.095
U 0.000
Q 0.095
Brier 0.050
Intercept 0.265
Slope 1.103
Emax 0.158
E90 0.012
Eavg 0.007
S:z -0.087
S:p 0.930
Ideal
Logistic calibration
Nonparametric
Actual Probability
Predicted Probability

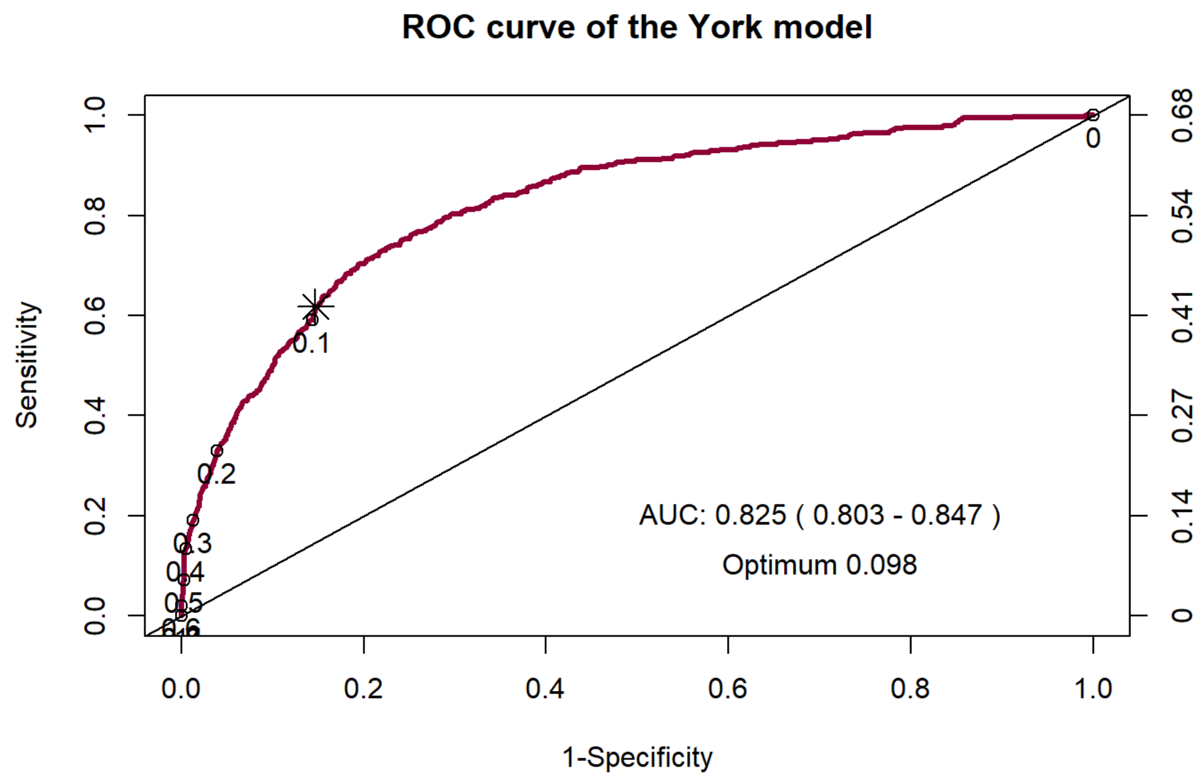
ROC curve of the York model
AUC: 0.825 (0.803 - 0.847)
Optimum 0.098
Sensitivity
1-Specificity
