# Supplementary material for: The Safety INdEx of Prehospital On Scene Triage (SINEPOST) study: The development and validation of a risk prediction model to support ambulance clinical transport decisions on-scene
Source: PLoS One. 2022 Nov 16;17(11):e0276515. doi: 10.1371/journal.pone.0276515 (PMC9668173; doi:10.1371/journal.pone.0276515)
Supplement: S6 Appendix — (PDF) [file pone.0276515.s006.pdf]

Appendix S6: Fair Machine Learning analysis

Fair machine learning is ensuring that any decision support (or making) prediction algorithm treats all individuals fairly and is not prejudiced. To evaluate whether the SINEPOST model is fair in a post-analysis, each individual group within a characteristic had their probability density plotted for comparison. If the model is fair, the distributions should look the same. in the analysis of fair machine learning, each category had the probability distributions mapped out along with the mean probability for the group. This was undertaken for age, gender, ethnicity and decile of deprivation.

Gender

Gender did demonstrate a difference in the probability distribution of transgender patients. However, there were only 8 transgender patients (1 classed as an avoidable ambulance conveyance) in the whole dataset and it is more likely that this is just a result of low sampling as opposed to a bias within the model.

Figure 1: Fair machine learning: Gender

Gender	grp.mean
Female	0.07
Male	0.07
Transgender	0.10
Unknown	0.06

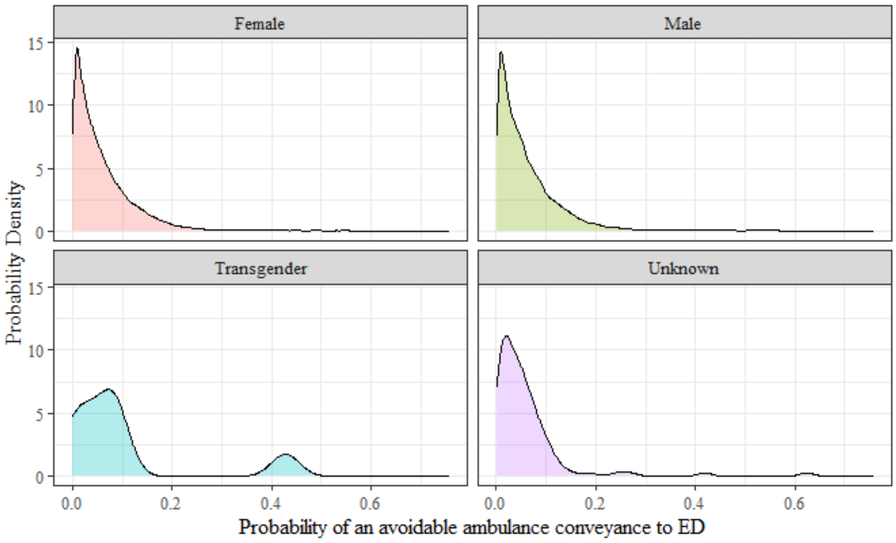

Age

For the purposes of making the analysis interpretable, age was categorised into groups. When age was initially left in as a candidate predictor, the distributions per age category differed significantly from each other and the younger age categories had higher predicted probabilities. This was not the case when age was removed as seen by figure 22. The SINEPOST model does not discriminate based on age.

Figure 2: Fair machine learning: Age

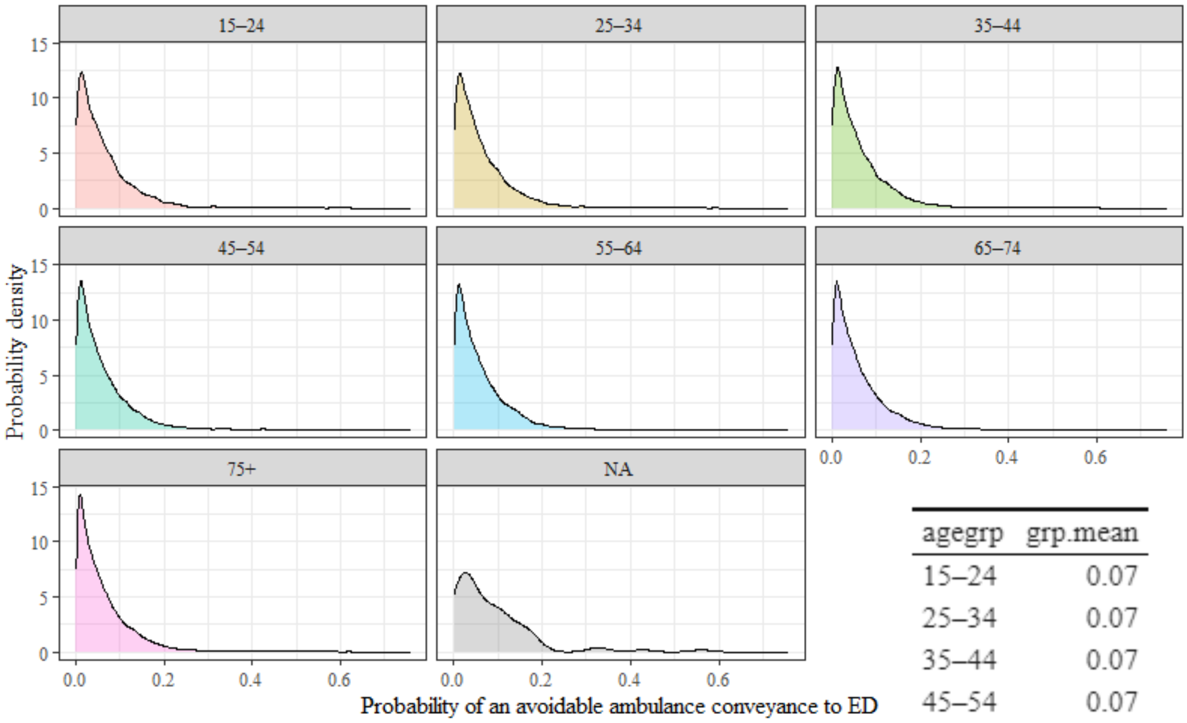

agegrp	grp.mean
15–24	0.07
25–34	0.07
35–44	0.07
45–54	0.07
55–64	0.07
65–74	0.07
75+	0.07
NA	0.08

Deciles of the Indices of Deprivation

The deciles of deprivation do not show any bias or discrimination between deciles. The probability distributions all appear similar with the only exception being the 'NA' category. Like the transgender category above, the 'NA' only had 180 instances which is small in comparison with the rest.

Figure 3: Fair machine learning: Indices of Deprivation

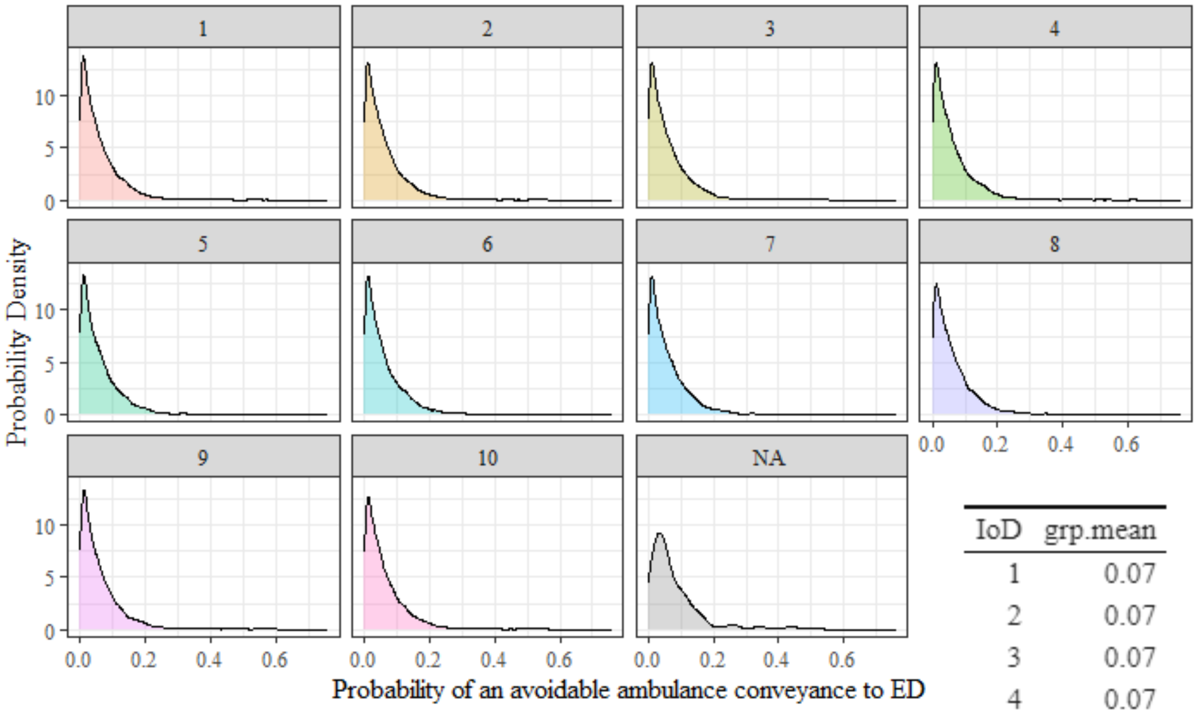

IoD	grp.mean
1	0.07
2	0.07
3	0.07
4	0.07
5	0.07
6	0.07
7	0.07
8	0.07
9	0.07
10	0.07
NA	0.08

Ethnicity

On initial modelling, the recursive feature elimination removed around two thirds of the ethnic categories. Due to this, it was decided to completely remove ethnicity to ensure the model was fair. On examining the distributions of the full model (figure 24) it appears that this was the right decision as there are no differences in the probabilities per ethnicity.

Figure 4: Fair machine learning: Ethnicity

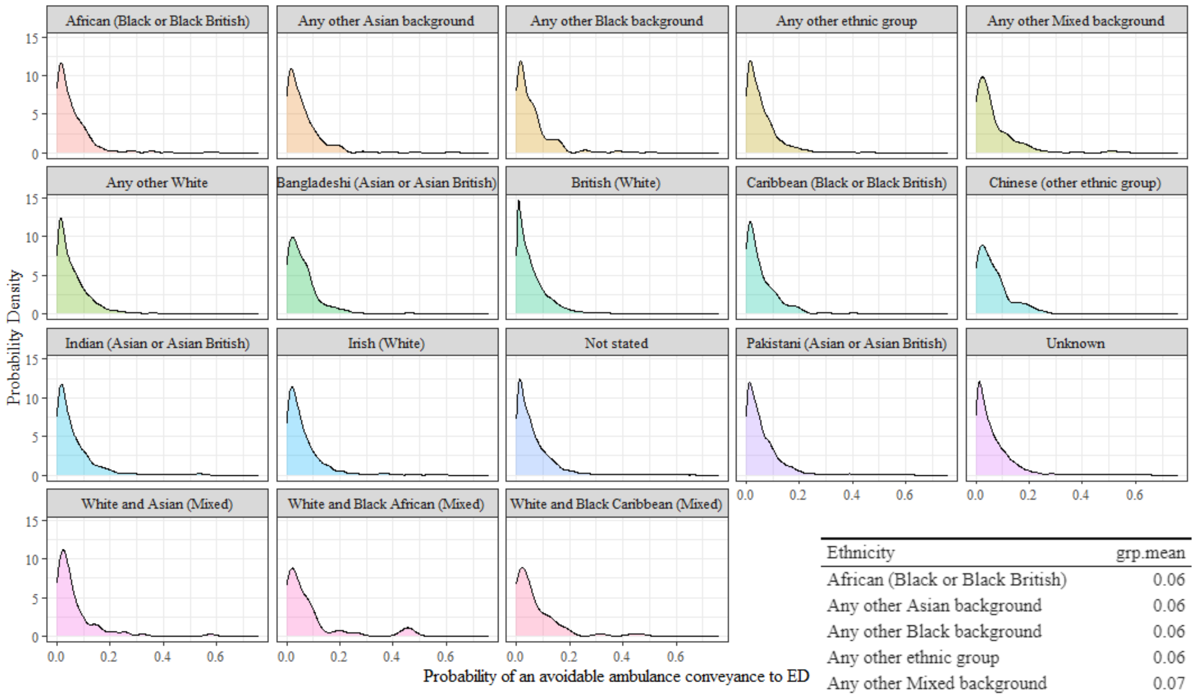

Ethnicity	grp.mean
African (Black or Black British)	0.06
Any other Asian background	0.06
Any other Black background	0.06
Any other ethnic group	0.06
Any other Mixed background	0.07
Any other White	0.07
Bangladeshi (Asian or Asian British)	0.06
British (White)	0.07
Caribbean (Black or Black British)	0.06
Chinese (other ethnic group)	0.06
Indian (Asian or Asian British)	0.06
Irish (White)	0.06
Not stated	0.07
Pakistani (Asian or Asian British)	0.06
Unknown	0.07
White and Asian (Mixed)	0.06
White and Black African (Mixed)	0.08
White and Black Caribbean (Mixed)	0.07
