# Supplementary material for: The Safety INdEx of Prehospital On Scene Triage (SINEPOST) study: The development and validation of a risk prediction model to support ambulance clinical transport decisions on-scene
Source: PLoS One. 2022 Nov 16;17(11):e0276515. doi: 10.1371/journal.pone.0276515 (PMC9668173; doi:10.1371/journal.pone.0276515)
Supplement: S7 Appendix — (PDF) [file pone.0276515.s007.pdf]

S7: All candidate variables from the ePCR dataset

Demographic variables

Variable name (units)	Values	Justification	Parameters	Cumulative
Age (Years)	18,19,20 years etc.	Age was the most significant predictive variable identified in the literature. Studies predicting higher-acuity outcomes such as critical care or hospitalisation have found that as patients become older the risk is greater.[107,109–113,133,165,166,168,169,171–175]	1	1
Gender	Male, Female, Transgender, Unknown	Studies are inconclusive as to whether gender is a predictor of acuity. Therefore there is a benefit to including it in this model. [172] [167] [170,171]	4	5
Ethnicity	Black, Asian, Mixed, White, Other	It has been shown to be associated with decisions to admit patients into the hospital. [168] [167]	5	10
Previous attendance within 24 hours	1,0	Fewer attendances within the last 12 months were a predictor of admission. [170]	1	11
Incident location	Care home, Domestic address, Not selected, Public place, School, Work, Other	Two studies have identified nursing home residency as a potential predictor for admission. [109] [168]	7	18
Social Deprivation (IMD)	1.1, 9.2, 13.4 …	The relationship between health and wealth is axiomatic with those more deprived having worse healthcare outcomes.	1	19

Social variables

Variable name (units)	Values	Justification	Parameters	Cumulative
GP address recorded	1,0	Social variables have rarely been used in prediction modelling. One example set in the USA used insurance type as a candidate predictor.[167] As alluded to in the background, patients present with complex physical, mental and social needs. To include social variables can support safe non-conveyance by ensuring there is an appropriate support network available. In the data, there are flags for if a GP, Next of Kin, parent, guardian or social worker is named in the ePCR.	1	20
NOK named	1,0		1	21
Parent named	1,0		1	22
Guardian named	1,0		1	23
Referral to service	Coroner, Police, Safeguarding adult, Safeguarding child …		5	28
Social worker named	1,0		1	29

Clinical variables

Variable name (units)	Values	Justification	Parameters	Cumulative
Primary survey: Catastrophic haemorrhage	1,0	This is the rapid assessment an ambulance clinician will do as they enter the scene to check if any life-threatening problems are present. It is reasonable to include these variables, as they are the earliest triaging that occurs on scene.	1	30
Primary survey: Cervical spine tenderness	1,0		1	31
Primary survey: Airway	Clear, Noisy, Occluded		3	34
Primary survey: Breathing	Normal, Abnormal, Not breathing		3	37
Primary survey: Pulse	Radial, Carotid, No palpable pulse		3	40
Primary survey: Level of response	Alert, Confusion, Verbal, Pain, Unresponsive		5	45
Mental capacity	1,0	This appears rational to include as mental capacity can have many different causes, and from a clinical perspective feature heavily in deciding if a patient can be left at home.	1	46
Clinical impression	Shortness of breath, Abdominal pain, Hypoglycaemia …	As described above when discussing chief complaint, studies have identified an association between clinical impression and acuity.	99	145
Initial pulse rate (bpm)	60,61,62 …	Many studies have used physiological observations as candidate variables for predicting acuity and they have shown great significance in final models.[110,112,113,167,169,171,176–178] These variables are often limited to pulse rate, respiratory rate, blood pressure, temperature, oxygen saturations, blood glucose and level of consciousness. The common principle in using them to predict critical care is that observational values that are extreme or deviated from the norm become highly	1	146
Initial respiratory rate (rpm)	16,17,18 …		1	147
Initial SpO2 (%)	96%,97%,98% …		1	148
Initial temperature (ºC)	36.2, 37.1, 37.5 …		1	149
Initial Systolic BP (mmHg)	120,121,122 …		1	150

Variable name (units)	Values	Justification	Parameters	Cumulative
Initial diastolic BP (mmHg)	80,81,82 …	predictive. Therefore, for predicting low acuity, it would be logical to include them as candidate predictors and expect the inverse relationship. That a normal physiological observation is predictive of a low acuity patient.	1	151
Blood glucose (mmol/L)	4.1, 5, 5.2, 10.1		1	152
Initial Glasgow Coma Scale (GCS) score	15,14,13,12,11,10,9,8,7,6,5,4,3	Level of consciousness is usually measured in adults using the Glasgow Coma Scale (GCS). In one study, the GCS was abbreviated to a simplified consciousness score (SCS). In regards to variable importance, it was ranked 1st across all learners.[112]	13	165
Initial GCS: Eye component	4,3,2,1		4	169
Initial GCS: Verbal component	5,4,3,2,1		5	174
Initial GCS: Motor component	6,5,4,3,2,1		6	180
Initial NEWS score	1,2,3 …	The National Early Warning Score (NEWS) is a composite scoring system based on respiratory rate, the saturation of oxygen in the blood (SpO_2), pulse rate, systolic blood pressure, body temperature and level of alertness. A copy of the latest version (NEWS2) can be found in appendix F. [179,180] Studies have shown there is a clear relationship between the NEWS score and patient acuity. [133]	1	181
Initial pain score	1,2, 3...	Pain has been a significant predictor for admission of patients from the ED. A study using natural language processing of free text fields in hospital documentation found that the most frequently used terms for admission were: pain, soreness and ache.[167]	1	182
Hypercapnic respiratory failure	1,0	There are observations that are present in DS2, but not necessarily captured in previous studies. This presents itself as an opportunity to explore potential new candidates and further contribute new knowledge in this area. Pupil size is a neurological observation that includes the size of the	1	183

Variable name (units)	Values	Justification	Parameters	Cumulative
initial pupil size left	1,2,3 …	person's pupil in the eye, and the reactivity of it. Peak flow is a measurement of force during exhalation and is used before and after treating a patient with respiratory conditions in order to assess effectiveness of treatment. A related variable is whether a person has hypercapnic respiratory failure. This is a relatively new field and is assessing whether a person has an abnormal respiratory physiology as part of their medical history, which would alter what their normal SpO_2 should be. An example would be a patient with COPD who may live with reduced SpO_2, and therefore the target % oxygen should also be lower.	1	184
initial pupil size right	1,2,3 …		1	185
Initial pupil reaction left	1,0		1	186
initial pupil reaction right	1,0		1	187
Subsequent pulse rate	60,61,62 …	The subsequent observations were included for two reasons. The first is that abnormal observations in subsequent recordings is important information. But also, the subsequent observations can be used to create observation intervals (the difference) which can be used as a predictor of deterioration.	1	188
Subsequent respiratory rate	16,17,18 …		1	189
Subsequent SpO2	96%,97%,98% …		1	190
Subsequent temperature	36.2, 37.1, 37.5 …		1	191
Subsequent Systolic BP	120,121,122 …		1	192
Subsequent diastolic BP	80,81,82 …		1	193
Subsequent Responsiveness	Alert, Confusion, Verbal, Pain, Unresponsive		5	198
Subsequent Glasgow Coma Scale (GCS) score	15,14,13,12,11,10,9,8,7,6,5,4,3		13	211
Subsequent GCS: Eye component	4,3,2,1		4	215
Subsequent GCS: Verbal component	5,4,3,2,1		5	220
Subsequent GCS: Motor component	6,5,4,3,2,1		6	226

Variable name (units)	Values	Justification	Parameters	Cumulative
Subsequent NEWS score	-1,0,1 …		1	227
Subsequent peak flow	300,301,302 …		1	228
subsequent pupil reaction left	1,0		1	229
subsequent pupil reaction right	1,0		1	230
subsequent pupil size left	1,2,3 …		1	231
subsequent pupil size right	1,2,3 …		1	232
Subsequent pain score	1,2, 3...		1	233
Difference pulse rate	-1,0,1 …	As discussed above, these are useful in identifying a deteriorating patient.	1	234
Difference respiratory rate	-1,0,1 …		1	235
Difference SpO2	-1,0,1 …		1	236
Difference temperature	-1,0,1 …		1	237
Difference Systolic BP	-1,0,1 …		1	238
Difference diastolic BP	-1,0,1 …		1	239
Difference Responsiveness	-1,0,1 …		1	240
Difference Glasgow Coma Scale (GCS) score	-1,0,1 …		1	241
Difference NEWS score	-1,0,1 …		1	242
Difference pain score	1,2, 3...		1	243
Difference peak flow	300,301,302 …		1	244

Variable name (units)	Values	Justification	Parameters	Cumulative
difference pupil reaction left	1,0		1	245
difference pupil reaction right	1,0		1	246
difference pupil size left	1,2,3 …		1	247
difference pupil size right	1,2,3 …		1	248
Abnormal ECG on primary	Left Bundle Branch Block, Right BBB, STEMI	No studies have used the initial abnormal ECG finding as a predictor variable in the past. The variable is found in the YAS ePCR and is an early indicator of something wrong with the patient's heart. It is a categorical variable that only accepts ST elevation MI (STEMI) and bundle branch blocks, which are also an electrical problem with the heart.	3	251

Interventional variables

Variable name (units)	Values	Justification	Parameters	Cumulative
ECG monitored	1,0	Interventional candidate predictors utilise the massive benefit of using electronic healthcare records at the granular level. In DS2, there is a plethora of interventions captured in the data including investigations like an electrocardiogram (ECG). There are also fields detailing equipment used such as an airway device or immobilisation equipment. Treatments are also captured including which drug has been given. Being able to include these into the model as candidates has a tangible benefit as it could reveal which interventions make a difference to patient acuity.	1	252
Supplemental oxygen	1,0		1	253
ICN type	Intravenous, Intraosseous, None		3	256
Drug 1	Adrenaline 1:1000, Co-codamol 30/500, Diazepam …		100	356
Drug 2	Adrenaline 1:1000, Co-codamol 30/500, Diazepam …		100	456
Drug 3	Adrenaline 1:1000, Co-codamol 30/500, Diazepam …		54	510
Drug 4	Adrenaline 1:1000, Co-codamol 30/500, Diazepam …		25	535
Drug 5	Adrenaline 1:1000, Co-codamol 30/500, Diazepam …		17	552
Drug 6	Adrenaline 1:1000, Co-codamol 30/500, Diazepam …		16	568
Drug 7	Adrenaline 1:1000, Co-codamol 30/500, Diazepam …		14	582
Drug 8	Adrenaline 1:1000, Co-codamol 30/500, Diazepam …		12	594
Airway type	ETT, LMA, OPA, NPA…		10	604
Immobilisation	Scoop, Cervical collar, Extrication board …		9	613
Advice given	Wound care, bereavement, head injury		8	621
Mobility	Stretcher, walked, hoist		15	636
CPR	1,0		1	637
